# Assessment and Challenges of Ligand Docking into Comparative Models of G-Protein Coupled Receptors

**DOI:** 10.1371/journal.pone.0067302

**Published:** 2013-07-02

**Authors:** Elizabeth Dong Nguyen, Christoffer Norn, Thomas M. Frimurer, Jens Meiler

**Affiliations:** 1 Center for Structural Biology, Vanderbilt University Medical Center, Nashville, Tennessee, United States of America; 2 Novo Nordisk Foundation Center for Basic Metabolic Research, University of Copenhagen, Copenhagen, Denmark; 3 Novo Nordisk Foundation Center for Protein Research, University of Copenhagen, Copenhagen, Denmark; 4 Department of Pharmacology, Vanderbilt Program in Drug Discovery, Vanderbilt University Medical Center, Nashville, Tennessee, United States of America; 5 Department of Chemistry and the Institute for Chemical Biology, Vanderbilt University Medical Center, Nashville, Tennessee, United States of America; Medical School of Hannover, United States of America

## Abstract

The rapidly increasing number of high-resolution X-ray structures of G-protein coupled receptors (GPCRs) creates a unique opportunity to employ comparative modeling and docking to provide valuable insight into the function and ligand binding determinants of novel receptors, to assist in virtual screening and to design and optimize drug candidates. However, low sequence identity between receptors, conformational flexibility, and chemical diversity of ligands present an enormous challenge to molecular modeling approaches. It is our hypothesis that rapid Monte-Carlo sampling of protein backbone and side-chain conformational space with Rosetta can be leveraged to meet this challenge. This study performs unbiased comparative modeling and docking methodologies using 14 distinct high-resolution GPCRs and proposes knowledge-based filtering methods for improvement of sampling performance and identification of correct ligand-receptor interactions. On average, top ranked receptor models built on template structures over 50% sequence identity are within 2.9 Å of the experimental structure, with an average root mean square deviation (RMSD) of 2.2 Å for the transmembrane region and 5 Å for the second extracellular loop. Furthermore, these models are consistently correlated with low Rosetta energy score. To predict their binding modes, ligand conformers of the 14 ligands co-crystalized with the GPCRs were docked against the top ranked comparative models. In contrast to the comparative models themselves, however, it remains difficult to unambiguously identify correct binding modes by score alone. On average, sampling performance was improved by 10^3^ fold over random using knowledge-based and energy-based filters. In assessing the applicability of experimental constraints, we found that sampling performance is increased by one order of magnitude for every 10 residues known to contact the ligand. Additionally, in the case of DOR, knowledge of a single specific ligand-protein contact improved sampling efficiency 7 fold. These findings offer specific guidelines which may lead to increased success in determining receptor-ligand complexes.

## Introduction

Being able to model the complex interactions between receptors and small molecule ligands offers immense opportunities for the basic biochemical understanding of signaling processes and for the development of pharmacological tool compounds and drugs that modulate receptor function. The human genome encodes for approximately 800 G-protein coupled receptors (GPCRs) that orchestrate the communication between a cell and its surroundings – an obvious place for small molecule drugs to interfere [1]. While more than 26% of our current small molecule drugs target Class A GPCRs alone [2], structure-based drug discovery has played a limited role in developing these molecules. GPCRs have been the subject of many structural, comparative modeling and docking studies. However in many cases, models are affiliated with high uncertainty and inaccuracy. Primary reasons include a lack of adequate template structures, the existence of multi-conformational states which require intense conformational sampling of not only the protein side chain but also backbone conformational space, in combination with the large variety of ligands that interact with GPCRs, including very flexible molecules which are notoriously challenging subjects for accurate docking [3], [4]. Nevertheless, increasing the availability of reliable GPCR models for structure-based drug discovery would be beneficial in the development of novel, potent and subtype-selective molecules. Since the landmark publication by Rasmussen et al. in 2007 [5], the number of experimentally determined GPCR structures has been increasing rapidly and now totals to 18 distinct GPCR structures that are recorded in the Protein Data Bank (PDB). While this is still just a small subset of the GPCR space, it provides a more substantial basis for comparative modeling and docking simulations.

Despite the increase in experimental structural information, it remains difficult to predict ligand-binding conformations in comparative models of GPCRs for all except the receptors most similar to those which have been solved experimentally [3], [4], [6]. This difficulty originates in part from the necessity of sampling both receptor and ligand flexibility which, due to the necessarily approximate nature of the force fields and protein-less/ligand-less sampling methods, results in the sampling of biologically irrelevant conformations. This complicates discrimination between the global minimum energy conformation (GMEC) and the local minimum energy conformations (LMEC) of the binding complex, as deeper sampling reveals many different energy-equivalent binding modes. The reason for the difficulty in GMEC and LMEC discrimination is, as discussed by Fleishman and Baker [7], related to the small energy gap in ligand binding, which moreover is challenging to measure as it is often mediated by polar contacts and water molecules.

For GPCRs, the ligand docking problem is even more difficult for three reasons. Firstly, the alignment is not trivial, as the transmembrane helices occasionally contain bulges and kinks and the length of the transmembrane helix is not conserved. Secondly, GPCRs are able to assume multiple different conformations with approximately the same energy, as demonstrated by studies on the beta-adrenergic receptors [8], [9]. Thirdly, three extracellular loops must be modeled, as they often contact the ligand and are involved in ensemble stabilization in some receptors [10], [11].

At the same time, improved algorithms and high-performance computing revolutionize our ability to sample protein conformational space swiftly, enhancing the possibility to accurately dock ligands into comparative models [12]. This, combined with the increasing number of available templates, lets us assess the applicability of rapid Monte Carlo Metropolis (MCM) sampling as implemented in the Rosetta suite of programs for GPCR comparative modeling and docking [13]. Specifically, we address the accuracy of backbone placement in transmembrane and extracellular loops, sampling of ligand binding modes and side-chain conformations in the binding site, and strategies to select accurate models from the large conformational space sampled.

## Methods

### Database Generation

**Figure 1:**
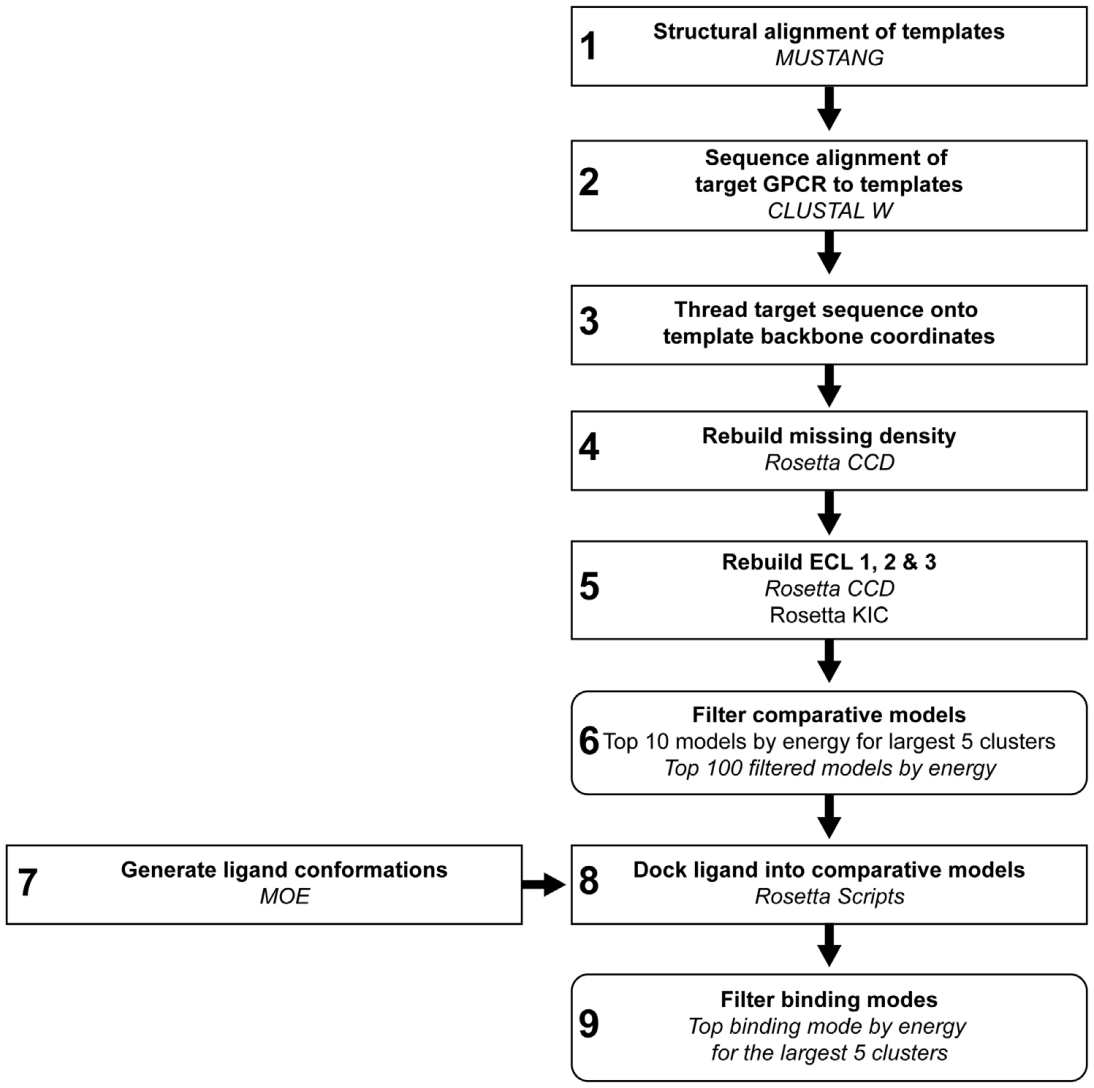
Flowchart of the comparative modeling and ligand docking protocol. For each step in the protocol, the name of the application or method used to execute each step is included. Where multiple methods are mentioned, the results from the method in italics were carried on to the next step.

The highest resolution experimental structure for each unique GPCR in the Protein Data Bank (PDB) at the time of writing was chosen for comparative modeling and ligand docking, as shown in Table 1. This includes the following G-protein coupled receptors: rhodopsin [14], β1-adrenergic [15], β2-adrenergic [16], A2A adenosine [17], CXCR4 chemokine [18], dopamine D3 [19], histamine H1 [20], S1P1 sphingosine 1-phosphate [21], M2 muscarinc acetylcholine [22], M3 muscarinic acetylecholine [23], mu-opioid [24], kappa-opioid [25], N/OFQ opioid [26] and delta-opioid [27]. Comparative models were constructed of each GPCR using the other 13 structures as templates. Ligand docking was performed with the small molecules crystallized within each receptor (Figure S1). A flowchart demonstrating the full protocol carried out in this study is shown in Figure 1. Full command lines for each step are included in (File S1).

**Table 1:** G-protein coupled receptor experimental structures and their ligands used in this study.

Protein name	PDBID/Chain	Loop length ECL1/2/3^a^	Resolution (Å)	Ligand	Rotatable bonds	Fold decrease in sampling efficiency when usingligand conformers b	Waterswithin 4Å c	Contacts(pocket/loops) d
Rhodopsin (bRh)	1U19/A	5/27/7	2.2	Retinal	5	4.0	1	19/6
Beta1-Adrenergic (B1Ar)	2VT4/B	6/26/6	2.7	Cyanopindolol	6	3.2	0	19/2
Beta2-Adrenergic (B2Ar)	2RH1/A	5/26/6	2.4	Carazolol	6	4.6	0	18/2
A2A adenosine (A2Ar)	3EML/A	6/25/8	2.6	ZM241385	4	15.0	7	13/5
CXCR4 chemokine (CXCR4)	3ODU/A	5/18/7	2.5	IT1t	7	68.1	7	7/6 (+1N term)
Dopamine D3 (D3R)	3PBL/A	8/16/7	2.89	Eticlopride	7	6.6	N/A	20/2
Histamine H1 (H1R)	3RZE/A	6/17/5	3.1	Doxepin	3	3.1	N/A	18/1
S1P1 sphingosine 1-phospate (S1P1R)	3V2W/A	10/18/8	3.35	ML056	11	42.4	N/A	21/5 (+2N term)
M2 muscarinic acetylcholine (M2R)	3UON/A	7/17/6	3	3-quinuclidinyl-benzilate	5	6.4	2	18/1
M3 muscarinic acetylcholine (M3R)	4DAJ/A	7/17/7	3.4	Tiotropium	5	6.4	N/A	19/1
Mu-opioid (MOR)	4DKL/A	7/20/6	2.8	β-FNA	8	27.2	4	15/0
Kappa-opiod (KOR)	4DJH/A	5/22/4	2.9	JDTic	7	8.8	5	22/2
N/OFQ opioid (NOP)	4EA3/A	4/19/6	3.01	C-24	8	50.6	1	17/4
Delta-opioid (DOR)	4EJ4/A	4/19/7	3.4	Naltrindole	2	1.6	N/A	15/0

Data collected from the Protein Data Bank.

a Loop lengths determined by DSSP.

b Ligand conformers generated by MOE. The fold decrease in sampling efficiency is the uniform sampling efficiency within a 2.0 Å radius (USE2.0) for the bioactive ligand conformation divided by USE2.0 for ligand conformers.

c Number of water molecules bridging the receptor and ligand within 4 Å of the ligand crystallized in the binding pocket.

d Number of residues in the receptor in contact with the ligand. Indicated is the number of contacts within the transmembrane region of the receptor binding pocket versus the loop or N-terminal regions.

### Sequence Alignment and Threading

The first step in constructing the models was performing a sequence alignment of the target sequence with a structural alignment of the other 13 GPCRs to be used as templates. A structure-based sequence alignment was generated of all 14 GPCR templates using MUSTANG [28] as seen in Figure S2 (Figure 1, Step 1). The sequence of the target GPCR was then aligned with the profile of structurally aligned templates using CLUSTALW [29] (Figure 1, Step 2). The sequence of the target GPCR was then placed onto the helical backbone coordinates of each template structure (Figure 1, Step 3). Any missing density and variable loop regions were constructed using the *ab initio* cyclic coordinate descent protocol in Rosetta [30], [31] (Figure 1, Step 4).

### Building in Missing Density and Extracellular Loop Regions in the Comparative Models

Missing density in the threaded models due to gaps or insertions in the sequence alignment were built in Rosetta using Monte Carlo Metropolis (MCM) fragment replacement combined with cyclic coordinate descent loop closure [30], [31] (Figure 1, Step 4). Cyclic coordinate descent (CCD) was inspired by inverse kinematic applications in robotics and closes loops by minimizing the sum of the squared distances between three backbone atoms of the moving N-terminal anchor and the three backbone atoms of the fixed C-terminal anchor through the adjustment of dihedral angles. Its speed and its ability to close a loop over 99% of the time gives CCD an advantage over other loop closure methods. In brief, loop regions defined by the user are chosen in a random order and for each loop, φ-ψ angles of backbone segments from homologous sequence fragments from the PDB, excluding those from the target experimental structure, are introduced into the loop regions. After the fragment substitution, small movements in the φ-ψ angles are performed to close breaks in the protein chain. After each defined loop has been closed, resulting full sequence models were subjected to eight iterative cycles of side chain repacking and gradient minimization of φ, ψ, and χ angles using the Rosetta scoring function with an implicit membrane potential [32]. A total of 200 models were constructed with each threaded model and the lowest energy model was chosen for a full remodeling of the extracellular loops (Figure 1, Step 5). Extracellular loops, as shown in Figure S3, were extensively rebuilt using both the cyclic coordinate descent loop closure method described above and the kinematic loop closure method described below. Approximately 1000 models were built for each target-template pair, resulting in a minimum of 13,000 comparative models per target structure.

A limited benchmark over the comparative modeling of six GPCRs was performed to compare the results of the kinematic loop closure (KIC) method in Rosetta [33] with CCD. KIC analytically determines all mechanically accessible conformations for six pivot torsion angles of a peptide chain using polynomial resultants. During kinematic loop closure, all mechanically accessible conformations for φ and ψ dihedral angle torsions from the first, middle and last residues in a loop segment, designated as pivot torsions, are sampled. The remaining torsion angles are randomly sampled using Monte Carlo minimization from Ramachandran probabilities of each amino acid. The six pivot torsions are solved analytically to close the loop. The protocol is performed for 720 rounds of high resolution loop closure and models accepted by the Metropolis criterion are subjected to side chain repacking and gradient minimization as described above. The data from the benchmark set comparing the two methods indicated that overall, CCD produced comparative models with an average root mean square deviation (RMSD) of 2.0 Å over extracellular loops (ECL) 1 and 3, which was significantly lower than the average RMSD over ECL1 and 3 for KIC at 2.6 Å (Table S1). The difference between CCD and KIC for the average RMSD over the full receptor was even more striking at 3.8 Å and 6.9 Å respectively. Results from CCD loop modeling were consequently used for further analysis.

### Selection Methods of Comparative Models for Docking

Comparative models were filtered for ligand docking using two different methods (Figure 1, Step 6). Both methods partially build on the observation that receptor accuracy is correlated with the Rosetta energy function (Figure S4). The first method was based on clustering of the 10% best scoring structures. Clusters were determined based on pairwise RMSD of all C-alpha atoms using bcl::Cluster [34] and a cluster radius of 3.0 Å. The best scoring models in each of the clusters were used for further analysis. The second method was created to avoid sampling of non-native ligand binding pocket conformations. Pocket residue positions were defined across all GPCRs as positions in the sequence alignment where C-alpha atoms of the residues had a distance of less than 4.0 Å to the ligand in at least one experimental structure. This yielded a list of 29 residue positions, which was reduced to 25 residue positions when the four residue positions at the top of transmembrane helices (TM) two and five were removed to avoid bias from structural alignment of the proteins. Pocket residues are shown in the alignment in Figure S2. Comparative models passed the filter only if C-alpha atoms of all pocket residues had an alignment equivalent pocket residue in another GPCR within a distance of a residue position specific cutoff. The cutoffs were chosen to be residue specific to represent varying flexibility in different parts of the receptor. The maximum distance between a specific pocket residue in any receptor and an equivalent pocket residue in any other GPCR, according to the alignment shown in Figure S2, was chosen as a distance cutoff for that particular residue position. When applying the knowledge-based filter, the self-experimental structure was not considered to avoid circular bias.

### Generation of Ligand Conformers

In preparation for docking, ligand conformers were generated by MOE (Molecular Operating Environment, Chemical Computing Group, Ontario, Canada) using the MMFF94x force field and Generalized Born implicit solvent model (Figure 1, Step 7). Conformers were generated using 10,000 iterations of the Low Mode MD method [35] with a redundancy cutoff of 0.25 Å. Energy cutoffs for ligand conformers were dependent on the number of rotatable bonds: 3 kcal/mol for 1–6 rotatable bonds, 5 kcal/mol for 7–9 rotatable bonds and 7 kcal/mol for 10–12 rotatable bonds [36]. The RMSD distribution for the generated ligand conformers compared to the bioactive ligand conformation is shown in Figure S10.

The ligand conformers were protonated as shown in Figure S1. These protonation states were determined based on the local environment in the individual experimental structures. In the case of ligand C-24, the protonation state is not what would be predicted without information from the experimental structure. We note that this adds some bias to the method. Likewise, the stereochemistry of the ligand (*E)*-IDT in CXCR4 was taken directly from the experimental structure. Of note, the experimental structure was solved with a mixture of (E) and (Z)-form, which cannot be clearly distinguished from the electron density (Raymond Stevens, personal communication).

### Docking Ligands into a Chosen Ensemble of Comparative Models

Ligand docking into the comparative models was performed with Rosetta Scripts [37]–[40] (Figure 1, Step 8). Each ligand was allowed to sample binding modes in a 5.0 Å radius from the coordinate representing the center of the ligand binding mode as given in the experimental structure. This adds some bias to the sampling, as the smallest unbiased docking sphere enclosing all ligand binding conformations has a radius greater than 5.0 Å. During the low-resolution phase of docking, rigid body orientation of the ligand centroid is performed through translation until the geometric center of the ligand is in a position not occupied by atoms in the receptor. High-resolution docking then begins with 1000 cycles of full rotational freedom until the attractive and repulsive forces fall below a threshold value. Six cycles of side-chain rotamer and ligand conformer sampling are then coupled with 0.1 Å, 0.05 radian ligand movements simultaneously in a Monte Carlo simulated annealing algorithm. All rotatable bonds within the ligand, except for planar conjugated bonds, were allowed full flexibility as indicated within the ligand parameters file. Ligand conformers are randomly chosen until the Monte Carlo criterion has been satisfied. A final minimization combines side-chain rotamer sampling with backbone torsion angle minimization with harmonic constraints on the C-alpha atoms.

The energy function used during the docking procedure contains terms for van der Walls attractive and repulsive forces, statistical energy derived from the probability of observing a particular side-chain conformation in the PDB, hydrogen bonding, electrostatic interactions between pairs of amino acids, and solvation assessing the effects of both side-chain/side-chain interactions and side-chain/ligand interactions. For each ligand, over 2,000 docked complexes were generated and evaluated in comparison to the experimental ligand binding mode using RMSD to the heavy atoms.

### Assessing the Size of the Ligand Conformational Space

We propose a new measure to enable comparison of docking benchmark studies across targets and to test how the methods compare to random sampling – the uniform sampling efficiency (USE2.0). The proposed measure is equivalent to the sampling frequency of better-than-2.0-Å-RMSD-binding-modes that would occur by random sampling in a 5.0 Å docking sphere with no occluding protein, given a set of ligand rotamers and full rotational and translational freedom. To calculate USE2.0, each i rotamer of the N rotamers in the generated ligand ensemble was aligned to the experimental structure and rotated along its principal axes (φ,θ,ψ) using M (40) uniform spacings. For sampling to be uniform, a correction factor, Cφ, is needed to account for the fact that the number of ways of choosing θ, given φ, is proportional to the circumference of the circle that θ draws on the φ,θ sphere [41]. The translation distance that increased the RMSD to 2.0 Å was determined for each rotamer-rotation set r(i,φ,θ,ψ). USE2.0 was then determined as the fraction between the volume of space containing binding modes below 2.0 Å and sampled volume of the 5.0 Å (R) docking sphere.
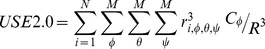



The distribution of RMSDs that arise from uniform sampling of ligand conformations is nontrivial, dependent on the ligand size and on the generated conformers. For the ligands considered in this dataset, USE2.0 varies from 10^−5^ for DOR to 10^−7^ for S1P1. The algorithm to determine USE2.0 is available in (File S2).

### Enrichment of Native-like Binding Modes using known Contacts between the Ligand and GPCR

When a mutation of a residue strongly affects ligand binding, this residue is often interpreted as having a direct contact to the ligand. To assess how this type of constraints enriches for the correct binding mode in our ligand-protein ensembles, we determined the average enrichment through 10,000 iterations of n randomly chosen known contacts with n running from 0 to all known contacts for a particular receptor-ligand interaction.

### Ligand-protein Evaluation through RMSD-based Clustering and Binding Energy

Results from the ligand docking study were evaluated using clustering on pairwise RMSD values calculated over the ligand heavy-atoms using bcl::Cluster with a 2.0 Å cutoff (Figure 1, Step 9). The lowest energy binding modes of the five largest clusters were chosen for further analysis. The coverage and accuracy of correct ligand-receptor contacts compared to the experimental structure was calculated on the top ranked models using SimiCon [42]. Contact coverage is calculated as the number of correct ligand contacts from the model divided by the total number of ligand contacts made in the experimental structure. Accuracy of correct contacts is calculated as the number of correct contacts divided by the total number of ligand contacts made in the model.

All plots were made with the Python 2D plotting library, matplotlib [43] and Prism 5.01 (GraphPad Software, San Diego, CA). The alignment figure was created used Aline [44]. Structural figures were created with PyMOL (PyMOL Molecular Graphics System, Version 1.5.0.4, Schrödinger, LLC).

## Results and Discussion

The results are presented in four parts. In the first part, we discuss the accuracy of comparative models generated by sequential sampling algorithms of the transmembrane and loop regions. Secondly, we discuss methods to select the most accurate models for ligand docking. Thirdly, we assess the equivalency of the local minimum interaction energy conformation (LMIEC) with the lowest energy that we sample and the experimental ligand binding mode and ask how much receptor flexibility can be sampled before the lowest energy LMIEC deviates from the experimental ligand-protein complex. In the fourth part, we assess the sampling efficiency of native-like ligand binding modes by docking the ligand ensemble into the ensemble of comparative models. Various methods used to identify native-like ligand binding modes in the resulting ensembles are explored.

### Templates of Higher Sequence Identity Produce More Accurate Comparative Models

**Figure 2:**
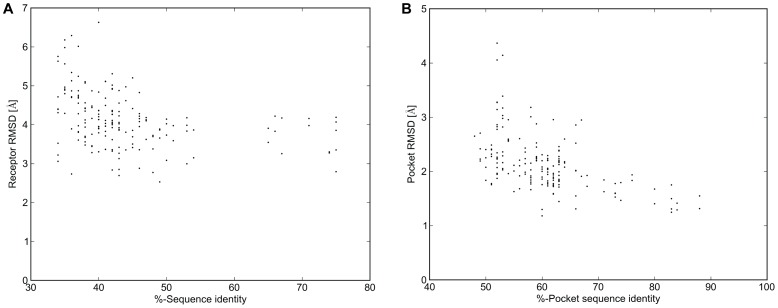
Template sequence identity versus comparative model RMSD. Each point represents the average RMSD over all comparative models of a target GPCR built using a particular template. For each target-template pair, percent sequence identity was calculated on the sequence alignment shown in Figure S2. Sequence identity is shown here to correlate with low average **A)** receptor RMSD, calculated over the C-alpha atoms of the full receptor and **B)** pocket residue RMSD, calculated over the C-alpha atoms of residues within the ligand binding pocket.

We generated 13,000 comparative models of each receptor through minimization and loop building in a sequential fashion as described in the Methods section. To assess the parameters which determine comparative model accuracy in the initial receptor ensemble, we considered the total energy of the models and the sequence identity of the template. Sequence identity was calculated on the aligned GPCR sequences as seen in Figure S2. As shown in Figure 2A, the average root mean square deviation (RMSD) of comparative models built with templates having greater than 50% sequence identity are consistently below 5.0 Å compared to the experimental structure. For residues in the ligand binding pocket (the pocket residues), the average RMSD of comparative models built with templates above 70% sequence identity within the pocket residues are frequently within 2.0 Å of the experimental structure (Figure 2B). In fact, only those targets with templates above 50% sequence identity were able to sample ligand binding pockets within 1.0 Å of the experimental structure (Figure S5).

As can be expected, this includes all target-template pairs within the same sub-family; for example, B1Ar and B2Ar serve as the best templates for each other at 74% sequence identity, as does M2R and M3R at 75% sequence identity. The β-adrenergic receptors also produced accurate models when used as templates for the muscarinic receptors at 48% sequence identity. The opioid receptors produced the most accurate comparative models when used as templates for each other at 65% to 75% sequence identity. In most cases, templates with high sequence identity also generate the lowest energy models in comparison to models based on other templates (Figure S5). Where there were exceptions, the lowest energy models were produced with templates with at least 45% sequence identity to the target receptor. Without a template having sequence identity above 50%, it continues to be difficult to get accurate models of the ligand binding pocket. While it was demonstrated that building multiple models based on different templates provides a better opportunity to sample the correct conformation and this is leveraged here [12], the generation of a smaller but improved conformational receptor ensemble could benefit from using multiple template structures in a single model [45]. Recently, Worth et al. 2011 demonstrated that similar or improved comparative models could be generated using a multi-template approach, where rotameric states as well as specific sequence and structural features could be modeled in light of the entire set of available experimental structures which otherwise might be absent when using a single template [46].

### Correct Helical Conformations are Recovered in Regions of Aligned Secondary Structure

C-alpha RMSD in comparison to the experimental structure was measured for the full receptor, transmembrane region and second extracellular loop (ECL2) region in the lowest energy models and the top ranked models by clustering (Table 2). Among the top ranked models for all 14 receptors, the transmembrane region was modeled with an average RMSD of 2.5 Å compared to their corresponding experimental structures. This average drops to 2.2 Å when considering only those models with template sequence identities above 50%.

**Table 2:** Benchmark results for comparative modeling of G-protein coupled receptors.

Protein name	Best sampled ECL2 Fullreceptor/TM region/ECL2RMSD (Å)	Lowest energy model Fullreceptor/TM region/ECL2RMSD (Å)	Top ranked model viaclustering^a^ Full receptor/TMregion/ECL2 RMSD (Å)	Percent of models with ECL2b under 2.0 Å^c^
BRh	4.2/1.3/3.3	4.7/2.5/6.2	4.1/1.4/7.5	0.4
B1Ar	2.8/1.6/2.9	3.7/1.7/4.5	3.2/1.2/4.3	53.9
B2Ar	3.4/1.4/2.7	3.2/1.9/5.9	3.7/1.7/4.4	45.2
A2Ar b	–	3.6/2.5/−	3.6/2.5/−	6.8
CXCR4	3.9/3.0/2.3	4.6/3.3/3.2	4.2/3.2/6.5	20.4
D3R	3.9/1.9/1.8	3.0/1.8/3.6	3.0/1.8/3.6	85.2
H1R b	–	1.6/2.5/−	2.4/2.4/−	0.8
S1P1R	3.3/2.0/3.4	5.8/2.1/4.8	3.6/2.0/5.4	0.6
M2R	2.2/2.4/1.9	2.2/2.3/4.2	2.2/2.3/4.2	7.0
M3R	3.1/2.4/2.3	3.1/2.9/5.3	2.7/2.4/5.2	9.7
MOR	4.7/3.1/1.6	3.6/1.9/4.6	2.4/2.8/5.8	14.6
KOR	3.7/2.6/2.7	4.4/2.5/6.7	3.1/2.9/5.8	3.8
NOP	2.2/2.6/2.6	3.2/2.8/6.6	3.0/2.4/5.8	10.5
DOR	2.0/2.8/2.5	3.3/2.2/5.5	3.3/2.2/5.5	10.6

Full receptor, transmembrane region and extracellular loop two RMSD over C-alpha atoms compared to the experimental structure is reported for models in each category.

a Top ranked model is determined by the lowest energy model from the largest cluster.

b ECL2 of A2Ar and H1R could not be evaluated because of unresolved structure in this region of the experimental structure in the Protein Data Bank.

c ECL2b represents the C-terminal half of ECL2, after the disulfide bond, which contains the residues that contribute to ligand binding as represented in the experimental structures from the PDB.

**Figure 3:**
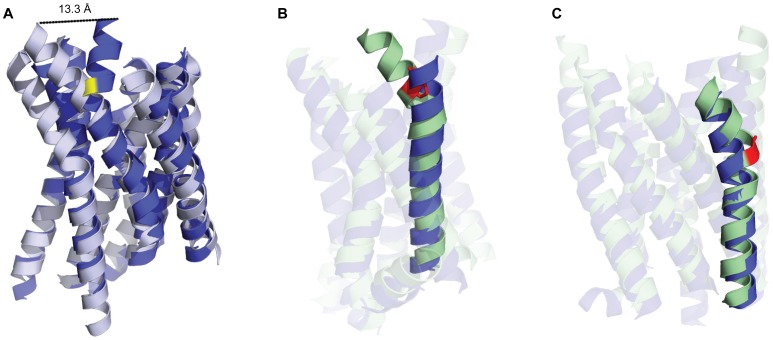
Structural representations of transmembrane helical regions from GPCR comparative models. **A)** TM7 in the top ranked comparative model of CXCR4 (blue) deviates from experimental structure (gray), specifically at W283 (highlighted in yellow). Cases where helical kinks exist in the template but are resolved in the comparative model include **B)** S1P1R, where the top ranked model (blue) resolves the kink in TM2 cause by P84 (highlighted in red) in the D3R template (green) and **C)** KOR, where the top ranked model (blue) resolves the kink in TM4 caused by G178 (highlighted in red) from the DOR template (green). The top ranked model is the best scoring model of the largest cluster, where clustering is performed on pairwise full receptor C-alpha RMSD over the top ten percent of comparative models by energy.

The maximum transmembrane region RMSD among top ranked models was seen for CXCR4 at 3.2 Å. In this case, helical placement of TM7 was shifted by six residues between the target sequence and the sequence of the templates, resulting in a gap in the alignment (Figure S2). Without reliable backbone coordinates to model the top of TM7, the resulting models rely on Rosetta to *de novo* fold the region using the CCD loop closure algorithm. The helical structure is recovered, but the top two helical turns of TM7 in the models are displaced from that of the experimental structure by distance of 13.3 Å (Figure 3A).

However, the conformation of the transmembrane helices is reasonably accurate throughout regions where transmembrane helices are aligned, specifically in terms of helical kinks. Deviations from ideal helical conformation are typically caused by proline or glycine residues and are important for both function and folding [47]. Major helical kinks occur in regions where proline residues are highly conserved between the GPCR sequences, particularly in TM 5, 6 and 7. In the two cases where templates had a proline or glycine-induced kink that was not present in the target, Rosetta was able to remove the kink and recover the correct conformation. The template of the top ranked S1P1R model, D3R, contains a proline at P84 which causes a kink in TM2 that was resolved by Rosetta (Figure 3B). The same is seen for TM4 in KOR, where the glycine-induced induced kink at G178 in DOR was removed during Rosetta energy minimization to recover the correct conformation (Figure 3C).

### Native-like Loop Conformations are Sampled but are Difficult to Identify by Score

Rebuilding the three extracellular loops was a point of focus during the modeling protocol because of their role in ligand binding. The first and third extracellular loops range in length from five to ten residues, which is within the range of successful loop prediction for Rosetta when applied on experimental structures [30], [31]. Here we find that the first and third extracellular loops are built with an average RMSD of 2.0 Å to the loop conformation from the experimental structures (Table S1). In several cases, identification of the correct loop conformation was possible using the energy of the loop (Figure S6, Figure S8).

**Figure 4:**
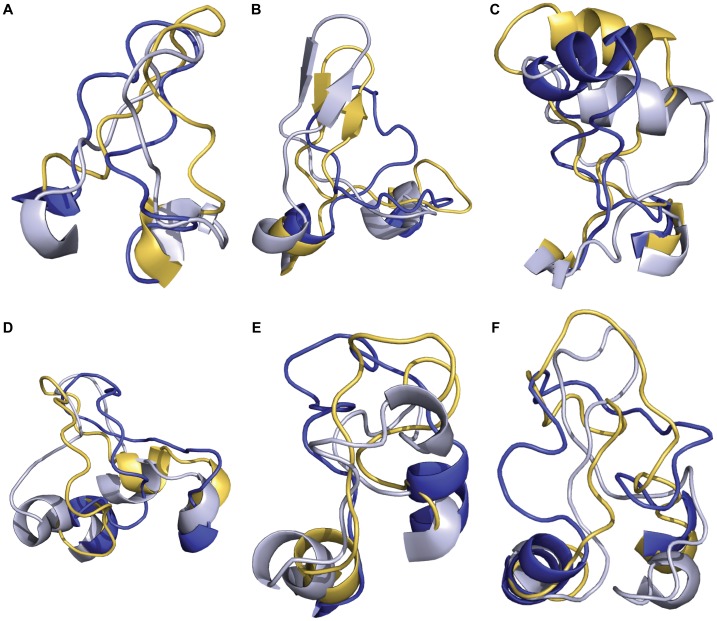
Structural representations of extracellular loop two from comparative models compared to experimental structures. For **A)** D3R, **B)** MOR, **C)** B1Ar, **D)** M2R, **E)** S1P1R and **F)** bRh, the experimental structure is represented in gray, the most accurately sampled model is represented in yellow and the top ranked model is represented in blue. The top ranked model is the best scoring model of the largest cluster, where clustering is performed on pairwise full receptor C-alpha RMSD over the top ten percent of comparative models by energy.

The second extracellular loop (ECL2) ranges in length from 16 to 31 residues. While the length of ECL2 is beyond the capability of prediction for loops excised from experimental structures, restriction of the sampling space was provided by requiring formation of the conserved disulfide bonds. The results demonstrate that ranking the most accurate ECL2 is difficult based on energy and clustering, since no top ranked models contained ECL2 RMSDs under 2.0 Å (Table 2 and Figure S7). However, it is possible to sample these native-like loop conformations, which is needed during docking to generate the correct ligand binding mode as observed in the experimental structure. Specifically, loop conformations were sampled below 2.0 Å for MOR, M2R and D3R (Table 2 and Figure 4). When focusing on the C-terminal region of ECL2, which is most often involved in ligand binding, we find native-like sampling for all models, with 0.4% to 85% of sampled ECL2 conformations below 2.0 Å (Table 2).

### Accurate ECL2 Conformations Often Recover Secondary Structure Elements

On average, the ECL2 RMSD for top ranked models by clustering was 5.3 Å, with the most accurate ECL2 conformations given for D3R at 3.6 Å, M2R at 4.2 Å and B1Ar at 4.3 Å (Table 2). Compared to the length of other ECL2s, which are about 21 residues long, D3R was relatively the easiest ECL2 to model with only 16 residues (Figure 4A). Other comparative models where ECL2 regions were most accurately sampled were those with secondary structure within the loop. Applying a fragment-based approach to *de novo* loop modeling allows for the insertion of secondary structure into loop regions where it is predicted from the sequence. In four of six cases, Rosetta was able recover helical elements found in ECL2 of experimental structures and one of five cases where β-sheets were found in ECL2 (Figure S9). MOR was the one case where β-sheets were conserved in the model, and the most accurately sampled ECL2 had an RMSD of 1.6 Å (Figure 4B). B1Ar (Figure 4C), B2Ar, M2R (Figure 4D) and M3R were the models in which helical elements were correctly sampled. In cases where predicted secondary structure in the target agrees with that of the template, such as with B1Ar and B2Ar, it would be beneficial to keep the loop conformation of the template and enforce the helical element [48]. The most difficult loop conformations to model were in S1P1R (Figure 4E) and bRh (Figure 4F), where the top ranked models only came within 5.4 Å and 7.5 Å of the experimental structure respectively. Both receptors have ECL2s longer than twenty residues with little secondary structure to stabilize the conformation. Additionally, ECL2 in both receptors packs against the N-terminal region, which was removed prior to comparative modeling. Therefore, inclusion of the N-terminal region into comparative modeling might be beneficial in these cases.

### Comparison with Previous Studies on GPCR Loop Modeling

Other studies have likewise addressed GPCR loop modeling. They include the protein local optimization program (PLOP) [48], [49], which samples amino acid rotamers in loop regions and evaluates models using a physics-based energy function while explicitly modeling membrane molecules. Modeller uses the CHARMM-22 force field and knowledge-based energy terms to optimize the loop conformation [50]. The algorithm employed by Nikiforovich et al. [51] performs geometric sampling of the loops using all possible conformations of the peptide backbone. In comparison to their study, Rosetta was able to rank loop conformations in comparative models more accurately than the loop conformations built *de novo* in experimental structures by Nikiforovich et al. [51]: bRh was modeled to 7.5 Å RMSD compared to 8.4 Å, B1Ar was modeled to 4.3 Å compared to 6.4 Å, and B2Ar was modeled to 4.4 Å compared to 7.4 Å. In their most recent study, Goldfeld et al. reported top ranked loop conformations built *de novo* in experimental structures as 2.7 Å for B1Ar and 2.2 Å for B2Ar [48]. However, the algorithm they used enforced the helical bounds within ECL2 for these structures. When considering the results from true *de novo* constructed loop conformations without the helical constraints, top ranked loop conformations from Rosetta are also more accurate than PLOP, whose top ranked ECL2 conformations were 9.1 Å for bRh, 5.6 Å for B1Ar and 13.8 Å for B2Ar.

These results indicate that even current state-of-the-art methods for loop modeling continue to have difficulty determining loop conformations, especially within comparative models. However, the experimental structures which we attempt to reproduce still only represent one of many possible loop conformations for these flexible regions and it is possible that more of the sampled conformations are in fact realistic [52], [53].

### Rosetta Captures Native-like Ligand Binding Pocket Conformations and Samples Beyond the Flexibility Evident from Experimental Structures

**Figure 5:**
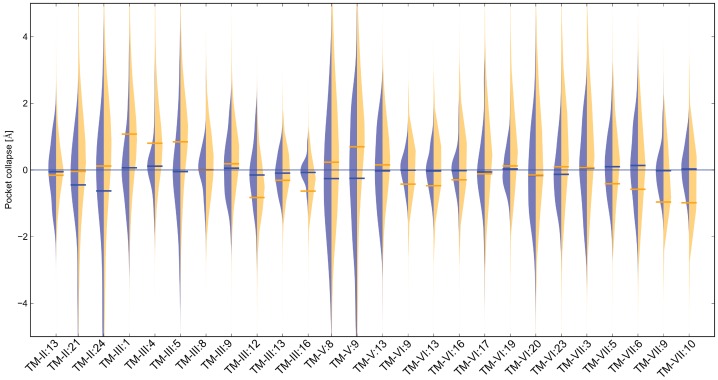
Ligand binding pocket flexibility within comparative models compared to experimental structures. For each of the 26 pocket residues in the ligand binding pocket of the receptor, pocket collapse is defined as the change in distance from each pocket residue to the ligand, measured between the model and the experimental structure. A positive pocket collapse value indicates that the pocket residue moves closer to the ligand in the model compared to the experimental structure, while a negative pocket collapse value indicates movement towards the receptor. The width of the beanplot area represents the number of models having a pocket collapse of a certain value for the threaded models (blue) and for all generated comparative models after loop rebuilding and energy minimization (orange), with corresponding blue and orange horizontal lines representing the average pocket collapse over the given set of models.

To assess the sampling density of residues lining the ligand-binding pocket, we aligned all the models to the experimental structures and measured the collapse of the pocket as the change in distance for each residue C-beta atom (C-alpha for glycine) to the closest ligand atom as determined from the experimental structures (Figure 5). The models display increased flexibility at the top of the transmembrane (TM) helices, as would be expected due to the variability represented by the crystallographic templates. With an average collapse of -0.1 Å and a standard deviation of 3.6 Å within all the comparative models generated, Rosetta samples beyond the flexibility that is represented by the experimental structures, which have an average collapse of -0.1 Å with a standard deviation of 1.0 Å (Figure 5). As the experimental structures are still a small and biased representation of the GPCR space, it is unclear if Rosetta is introducing too much flexibility in these regions. However, for the present study, many comparative models within our ensemble will not make constructive interactions with the ligand due to non-native placement of the residues involved in ligand binding.

### Knowledge-based Filters Improve the Accuracy of the Ligand-binding Pocket

**Figure 6:**
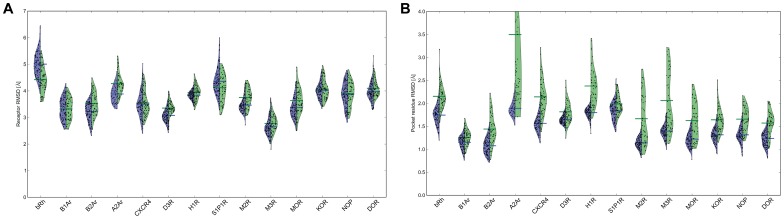
Comparison of two comparative models analysis methods: filtering by ligand binding pocket residues and clustering on RMSD. For each receptor, the ten lowest energy models of the largest five clusters are represented in green and the one hundred lowest energy models from the knowledge-based filter on residues in the ligand binding pocket are represented in blue. The width of the beanplot area represents the number of models having a particular **A)** receptor RMSD and **B)** pocket residue RMSD, with the corresponding horizontal lines representing the average RMSD for all models clustered by RMSD (green) and models from the knowledge-based filter (blue).

Because Rosetta samples the flexibility of the transmembrane region beyond the variability that is represented in the experimental structures, a knowledge-based filter was created which focused on the pocket residues alone to identify models that would be suitable for ligand docking. Models with structural deviation beyond the maximum flexibility observed within the binding pocket in existing experimental structures were removed, as described in the Methods section. The filter accepted between 0.2% and 10% of the models from the initial receptor ensemble and the overall RMSD of these models were comparable to those identified by traditional clustering methods (Figure 6A). For several receptors, there was a correlation between pocket RMSD and receptor energy (Figure S5) and based on this correlation, energy was used to reduce the filtered ensemble to a maximum of 100 structures. In this filtered ensemble, the receptor collapse was 0.3 Å with a standard deviation of 0.8 Å, which is slightly greater compared to what is seen in the experimental structures. This is possibly due to favorable energy when collapsing the pocket. The average RMSD of residues constituting the common ligand binding pocket is significantly improved compared to a receptor ensemble selected by clustering of the initial receptor ensemble (Figure 6B).

### Accuracy of the Ligand Conformer Ensemble is Highly Dependent on Ligand Flexibility

The generation of ligand conformations is not a trivial process, as the bioactive ligand conformation need not occupy its aqueous GMEC [36], [54], [55]. Our approach was to create ensembles of low energy ligand conformations (LMECs) and allow Rosetta to minimize these conformations in the context of a fully flexible receptor. Nevertheless, ligand ensembles will inevitably contain irrelevant conformations which results in the search of irrelevant binding modes. Low energy ligand conformations were generated with MOE and the energy cutoff was determined by the number of rotatable bonds within the ligand [36], as discussed in the Methods section.

To evaluate how the use of such ligand ensembles would affect sampling efficiency of ligand binding modes with RMSD below 2.0 Å, the Uniform Sampling Efficiency of binding modes below 2.0 2.0 Å (USE2.0) was calculated for all ligand ensembles and compared to that of the bioactive ligand conformation. While the uniform sampling efficiency dropped by only 6.0±3.8 fold for the majority of the ligands by using ligand conformers instead of the bioactive ligand conformation, it dropped by 68.1, 50.6, 42.4 and 27.2 fold for IT1t, C-24, ML056 and beta-FNA respectively (Table 1 and Figure S10). These ligands are characterized by many degrees of freedom, which contribute to the difficulty of sampling the bound conformation accurately.

While a benchmark of ligand ensemble generation methods was beyond the scope of this study, we noted some reduction in the number of non-native ligand conformers by using the Generalized Born implicit solvent instead of the distance-dependent dielectric constant. Further improvement might be possible in some cases by using MD-simulations to generate a canonical ligand ensemble weighted according to the Boltzmann distribution to identify the most populated and thereby most probable ligand conformations. For large, flexible ligands, a fragment-based docking approach might be more suitable and has already been applied in many drug design studies [56]–[58].

### Interaction Energy is not Reliable for Identification of the Experimental Ligand Binding Mode when Docking into Flexible GPCR Comparative Models

The bioactive ligand conformation from the experimental structure as well as ligand conformers generated by MOE were docked into the top ranked comparative models as evaluated by clustering and the knowledge-based filter. While Rosetta considers the energy of the receptor while sampling ligand binding poses, noise generated by the multiple loop conformations makes it difficult to identify low energy binding modes using the total Rosetta energy for the receptor-ligand complex. As a result, we choose to make the assumption that all structures from the comparative model ensemble have equal energy and accuracy when docking the ligands. Thus the local minimum interaction energy conformation (LMIEC) with the lowest energy that we sample needs to equate the energy of the experimental ligand binding mode in order to be useful for its identification. To test the extent to which this occurs, the bioactive ligand conformation was re-docked into the experimental structure it came from with no relaxation of the complex after docking (Figure S11). In 13 out of 14 cases we find that the lowest energy LMIEC was within 2.0 Å of the experimental ligand binding mode and that other LMIECs were significantly separated from the lowest energy LMIEC by 4.6±3.0 Rosetta Energy Units (REU). The one case where the lowest energy LMIEC deviated from the experimental ligand binding mode is IT1t in CXCR4, which has exceptionally few interactions to the protein, 7 water molecules within 4 Å, and contacts a residue in the N-terminal region, which is not represented in our models.

When repeating the protocol with the addition of flexibility within the receptor through a minimization step, the lowest energy LMIEC deviated from the experimental ligand binding mode in 6 out of 14 cases and with an insignificant energy gap of 0.3±2.1 REU, showing that lowest energy LMIEC is not suitable for identification of the experimental ligand binding mode in flexible models. However, while the ligand binding mode within a RMSD of 2.0 Å to the experimental structure could not be identified consistently by interaction energy in this highly biased analysis, it was possible to sample the correct binding mode in all 14 cases (Table 3).

**Table 3:** Sampling efficiency for ligand docking results.

Proteinname	Bioactive ligand docked to experimental structure, no minimization, n = 1000^1^	Bioactive ligand docked to experimental structure,minimized, n = 1000^1^	Bioactive ligand docked to topmodels fromknowledge-basedfilter, n = 2000^1^	Bioactive liganddocked to topmodels fromclustering byRMSD, n = 6000^1^	Ligand conformersdocked to topmodels fromknowledge-basedfilter, n = 10000^2^	Ligand conformersdocked to topmodels fromclustering byRMSD, n = 6000^2^
BRh	0.05 (5•10^4^)	0.04 (5•10^4^)	1.94 (7•10^2^)	2.40 (2•10^2^)	2.66 (5•10^2^)	3.52 (70)
B1Ar	0.03 (5•10^4^)	0.02 (5•10^4^)	2.05 (5•10^2^)	2.11 (4•10^2^)	2.18 (10^3^)	2.07 (10^3^)
B2Ar	0.03 (4•10^4^)	0.03 (4•10^4^)	2.19 (3•10^2^)	2.10 (3•10^2^)	2.27 (10^3^)	2.40 (8•10^2^)
A2Ar	0.08 (9•10^3^)	0.81 (2•10^3^)	2.85 (20)	2.92 (10)	3.22 (10^2^)	3.70 (30)
CXCR4	0.54 (10^4^)	0.96 (5•10^3^)	2.68 (10^2^)	2.62 (10^2^)	4.00 (3•10^2^)	ND* ^#^
D3R	0.04 (4•10^4^)	0.08 (3•10^4^)	2.22 (3•10^2^)	2.40 (2•10^2^)	2.59 (7•10^2^)	2.70 (6•10^2^)
H1R	0.01 (3•10^4^)	0.03 (3•10^4^)	2.07 (3•10^2^)	1.85 (5•10^2^)	2.17 (7•10^2^)	2.15 (8•10^2^)
S1P1R	0.05 (7•10^4^)	0.07 (7•10^4^)	3.30 (40)	2.40 (3•10^2^)	4.00 (4•10^2^)	3.30 (2•10^3^)
M2R	0.02 (4•10^4^)	0.02 (4•10^4^)	1.84 (6•10^2^)	2.38 (2•10^2^)	2.57 (7•10^2^)	2.74 (5•10^2^)
M3R	0.03 (3•10^4^)	0.03 (3•10^4^)	1.94 (4•10^2^)	2.26 (2•10^2^)	2.44 (8•10^2^)	2.74 (4•10^2^)
MOR	0.14 (2•10^4^)	0.35 (10^4^)	2.02 (3•10^2^)	2.27 (2•10^2^)	2.43 (3•10^3^)	3.40 (3•10^2^)
KOR	0.04 (2•10^5^)	0.03 (2•10^5^)	2.07 (2•10^3^)	2.55 (7•10^2^)	3.10 (2•10^3^)	2.92 (3•10^3^)
NOP	0.11 (5•10^4^)	0.11 (5•10^4^)	2.24 (4•10^2^)	2.15 (5•10^2^)	3.52 (10^3^)	3.70 (7•10^2^)
DOR	0.08 (2•10^4^)	0.07 (2•10^4^)	1.51 (8•10^2^)	1.77 (5•10^2^)	1.57 (10^3^)	1.82 (6•10^2^)

Reported is the negative log of the sampling efficiency of ligand binding modes within 2.0 Å RMSD of the bioactive ligand conformation within the experimental structure as measured over the ligand heavy-atoms.

* denotes where sampling efficiency of Rosetta is worse than the worst-case uniform sampling scenario.

# ND denotes not defined. No binding modes within 2.0 Å were sampled for this case.

1 fold improvement over USE2.0 of bioactive ligand is given in parentheses.

2 fold improvement over USE2.0 of ligand conformers is given in parentheses.

### Sampling of Native-like Ligand Binding Modes is on Average 10^3^ Fold Increased over Random Sampling

**Figure 7:**
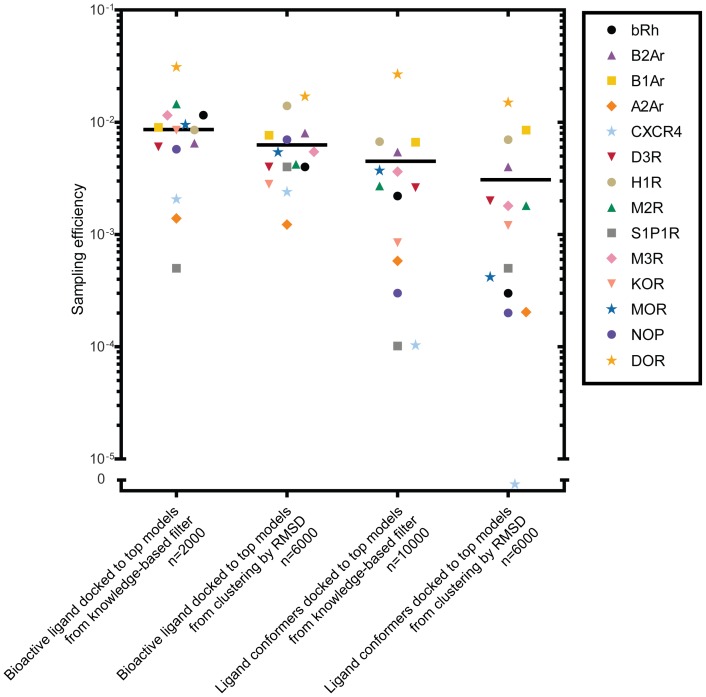
Sampling efficiency for docking into comparative models. For each receptor, the fraction of binding modes sampled within 2.0 Å of the experimental binding mode is represented for docking the bioactive ligand and ligand conformers into the top models chosen by the knowledge-based filter and clustering by RMSD. The average sampling efficiency for each method is represented by the black solid line.

Having generated receptor-ligand complexes through docking, we asked how frequently the experimental ligand binding mode was sampled within an RMSD of 2.0 Å and compared this to the sampling efficiency that can be achieved using uniform sampling in a 5.0 Å docking sphere with no protein (USE2.0). In the receptor ensemble that was selected based on the knowledge-based filter, we found that the experimental ligand binding mode was sampled in all cases with an average of 10^3^ fold increase over USE2.0 (Figure 7 and Table 3). The correct binding mode for S1P1R and CXCR4 was sampled correctly least often with only one correct binding mode out of approximately 10^4^ generated models. The reason for the difficulty of sampling the correct S1P1R ligand binding mode is most likely related to its flexibility and its contacts to the N-terminus, which is lacking in our models. The low number of ligand-protein contacts in the model seems to be the main reason for the poor sampling efficiency of IT1t in CXCR4, which as discussed above, was not in an interaction energy minimum when its bioactive conformation was docked into a backbone static receptor. Even so, sampling the experimental ligand binding mode within 2.0 Å RMSD was increased 300 fold over USE2.0 for both S1P1R and CXCR4, demonstrating preference of biologically relevant ligand-protein interactions during the docking procedure.

On the other end of the spectrum of sampling efficiency is DOR, which sampled the correct binding mode in 266 out of approximately 10,000 cases –10^3^ times better than USE2.0. The ligand in DOR, natrindole, has only 2 degrees of freedom, with all conformers below 1.0 Å of the bioactive ligand conformation (Table 1), and binds to the receptor mainly through hydrophobic contacts and one salt bridge. For all other cases, docking multiple ligand conformations into the comparative models sampled binding modes within 2.0 Å of the experimental binding mode less than 1% of the time (Figure 7).

### Sampling of Native-like Ligand Binding Modes Improve within the Knowledge-based Filtered Comparative Model Ensemble

To assess the effect of the knowledge-based filter we compared the sampling efficiency in models selected with the knowledge-based filter receptor ensemble with those chosen by traditional clustering methods and found that sampling efficiency is improved in 10 out of the 14 cases (Table 3 and Figure 7).

**Figure 8:**
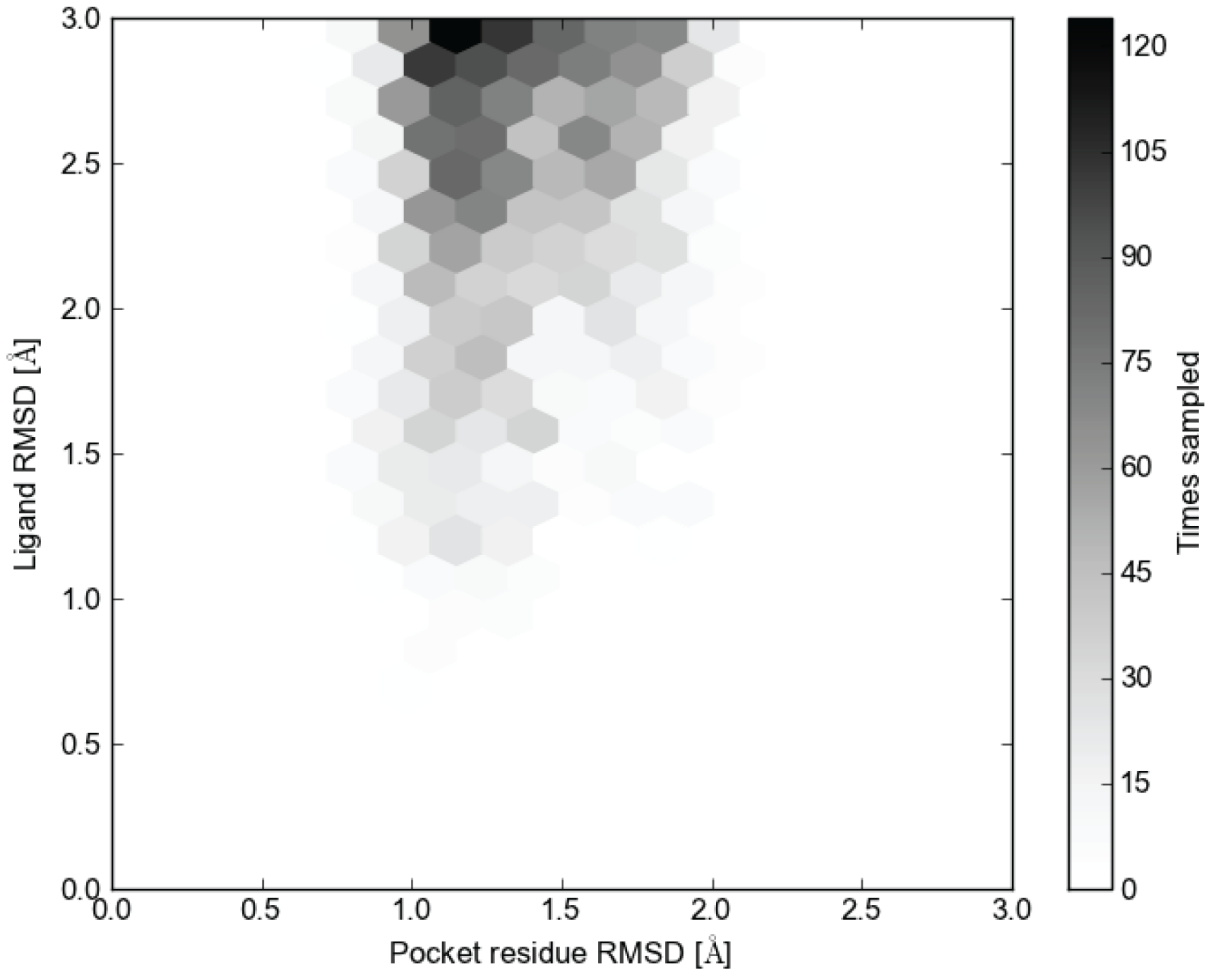
Sampling density of ligand binding modes versus pocket residue RMSD. The number of binding modes within the given RMSD of the experimental ligand binding mode is given for each pocket residue RMSD.

Additionally, as an attempt to identify which parameters of the receptor models yielded native-like ligand binding modes, we examined the importance of the pocket residue RMSD in Figure 8. Accuracy between the C-alpha atoms of the pocket residues does not guarantee accurate ligand placement, as side chain placement varies greatly and creates many non-native binding pockets. Also, given the flexibility of the ligand conformations, it is expected that the docking algorithm detects alternate binding modes within a particular binding pocket conformation. Despite this, we show that more accurate placement of the residues within the ligand binding pocket leads to more binding modes sampled within 2 Å of the experimental ligand binding mode using the knowledge-based filters and templates of high sequence identity (Figure 7, Figure S12). Importantly, while we show a correlation between pocket RMSD and ligand RMSD, the same effect cannot be shown when selecting receptor models using full receptor RMSD based clustering. This is likely due to the irrelevant noise that arises from non-pocket residues.

### Interaction Energy Enriches for Experimental Ligand Binding Modes

**Figure 9:**
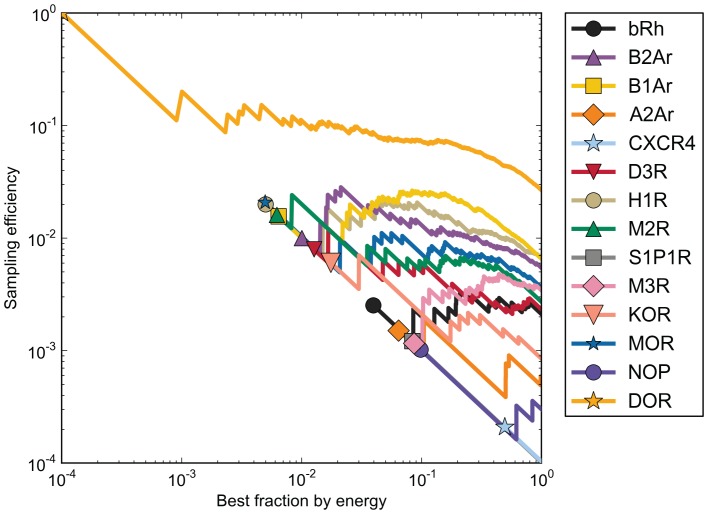
Enriching sampling efficiency with energy cutoffs. The sampling efficiency for binding modes sampled within 2.0 Å of the experimental binding mode at each fraction of comparative models selected by Rosetta interaction energy is presented for each receptor.

Despite the lack of robustness in the use of interaction energy to identify the correct binding modes in relaxed experimental structures, we expected that it might be useful for enrichment of correct binding modes in our docking ensembles by removing obvious non-fit ligand-protein interactions. We found that an enrichment of approximately three fold can be achieved in most cases by taking the 10% best scored structures, as shown in Figure 9. However, when taking the top 10% of structures for bRh, CXCR4 and M3, sampling efficiency dropped. There was no correlation between optimal cutoff value and the overall sampling efficiency or the size of the largest cluster (data not shown).

### Clustering Aids in Selecting Native-like Models

**Figure 10:**
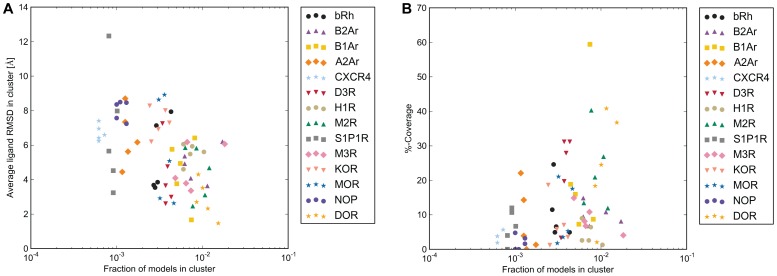
Clustering captures binding modes with lower RMSD and increased contact coverage. Binding modes for each receptor were clustered by ligand heavy-atom RMSD with a cutoff of 2.0 Å. When compared to smaller cluster sizes, the large cluster sizes were more likely to capture **A)** lower average ligand RMSD to the experimental binding mode and **B)** a higher percentage of correct ligand contacts. Contact coverage was calculated using SimiCon [42].

In spite of the low sampling efficiency of the experimental ligand binding modes, we hypothesized that clustering mediated through total energy optimization during docking might occur, and thus offer a method to identify native-like ligand binding modes. Notably, however, the Rosetta ligand docking algorithm does not in principle generate a Boltzmann distribution, but instead emphasizes sampling of rare binding modes, in hope of identifying a rare native-like global minimum interaction energy conformation [39]. This might blur any tendencies for clustering around an experimental ligand binding mode. Clustering was performed on the heavy-atom ligand RMSD with a cutoff of 2.0 Å and the lowest energy binding modes of the largest five clusters were examined further. Other cutoffs of 2.5, 3.0 and 3.5 Å, were also considered, but did not provide any improvement of clustering performance. The percentage of models in the largest clusters was below 1% for most receptors (Figure 10). For the receptors in which convergence occurred, however, there was some correlation between cluster size and ligand RMSD (Figure 10A).

**Figure 11:**
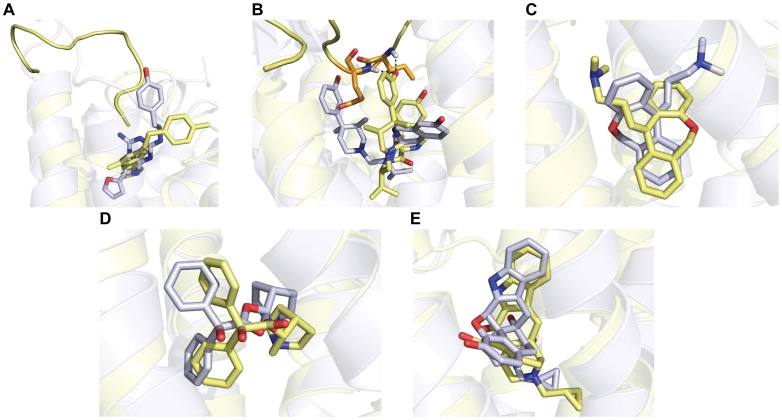
Structural representations of ligand binding modes compared to experimental structures. Incorrect loop placement and incorrect ligand orientation often prevent Rosetta from converging on the experimental ligand binding mode. Ligand binding modes from the experimental structures are shown in gray and the top ranked model via clustering by ligand RMSD is shown in yellow for **A)** A2Ar, **B)** KOR and **C)** H1R. Cases where top ranked binding modes captured the experimental binding mode within 2.0 Å were **D)** DOR and **E)** M2R**.**

Within the largest clusters for each receptor, there was on average 12% coverage and 10% accuracy of the correct contacts between the ligand and receptor (Table 4 and Figure 10B). For CXCR4, KOR and NOP, alternate modes are preferred over the experimental binding mode. In examining cases where the experimental binding mode is not preferred, several problems are identified which keeps the ligand from binding in the correct mode. For CXCR4 and NOP, less than 30% of the ligand conformers came within 2.0 Å of the bioactive ligand conformation, resulting in inaccurate docking results. For ligands binding high within the receptor binding sites such as A2Ar, incorrect loop placement in the models blocks the ligand from docking in the correct mode (Figure 11A). Incorrect loop placement can also induce hydrogen bonds favorable to the ligand which move it into an incorrect binding mode, as shown in KOR (Figure 11B). For many ligands, Rosetta places the ligand in the correct position but is unable to discriminate the correct interactions and flips the ligand orientation as seen in H1R (Figure 11C), indicating possible inaccuracies within the force field and improper treatment of polar interactions. There were two cases, DOR and M2R, in which Rosetta was able to identify the correct binding mode within 2.0 Å in the top ranked clusters (Table 4), shown in Figure 11D and 11E. Docking naltrindole in DOR and 3-quinuclidinyl-benzilate in M2R was simplified by the limited number of rotatable bonds in the ligand and high sequence identities of the templates.

**Table 4:** Top ranked binding modes for ligands docked into G-protein coupled receptor comparative models.

Protein name	Cluster Rank^a^	Ligand RMSD	Coverage of correct contacts^b^	Accuracy of correct contacts^c^
BRh	5	3.4	0.11	0.10
B1Ar	2	2.13	0.60	0.50
B2Ar	2	3.48	0.11	0.10
A2Ar	5	3.48	0.22	0.15
CXCR4	3	6.23	0.04	0.04
D3R	4	2.26	0.20	0.14
H1R	5	4.81	0.10	0.10
S1P1R	2	3.37	0.12	0.15
M2R	4	1.86	0.40	0.30
M3R	2	3.5	0.11	0.10
MOR	1	2.7	0.18	0.11
KOR	4	5.67	0.01	0.01
NOP	1	6.72	0.02	0.01
DOR	1	1.78	0.37	0.21

a The lowest energy binding mode from the largest 5 clusters, determined by heavy-atom ligand RMSD with a cutoff of 2 Å, was used for evaluation. Given here is the cluster rank for the lowest ligand RMSD of the top 5 ranked models.

b Coverage of correct contacts was calculated with SimiCon [42] and is the number of correct contacts divided by the total number of ligand contacts made in the experimental structure.

c Accuracy of correct contacts was calculated with SimiCon [42] and is the number of correct contacts divided by the total number of ligand contacts made in the model.

### Comparison with Previous Studies on Ligand Docking into GPCR Comparative Models

Using Glide [59] and Induced Fit Docking [60] to dock ligands within biased comparative models of GPCRs, Beuming and Sherman [6] ranked ligand binding modes within 2.5 Å of the experimental ligand binding mode in six out of the ten receptors they modeled. In these six cases, success was likely due to the structural similarity of the templates, which always came from receptors of the same sub-family: β-adrenergic receptors were used as templates for each other and for H1R and the muscarinic receptors were used as templates for each other. Alignments were manually refined to ensure correct alignment of loop regions and the disulfide bridge within ECL2. Only regions with missing density according to the alignment were rebuilt using PLOP [48]. Additionally, the ligand from the template structure remained within the model during the comparative modeling process, which may have assisted in the preservation of the ligand binding pocket. While remaining relatively unbiased in sequence alignment and *de novo* loop rebuilding, Rosetta was able to sample binding modes within 2.5 Å of the experimental ligand binding mode in all cases. However, inaccuracies in the energy function and flexibility introduced within the pocket residues made it difficult to identify native-like binding modes as top ranked. As discussed above, Rosetta had success in ranking the correct binding mode only in the cases of M2R and DOR.

### Sampling Efficiency is Increased by One Order of Magnitude for Every 10 known Ligand-protein Contacts

**Figure 12:**
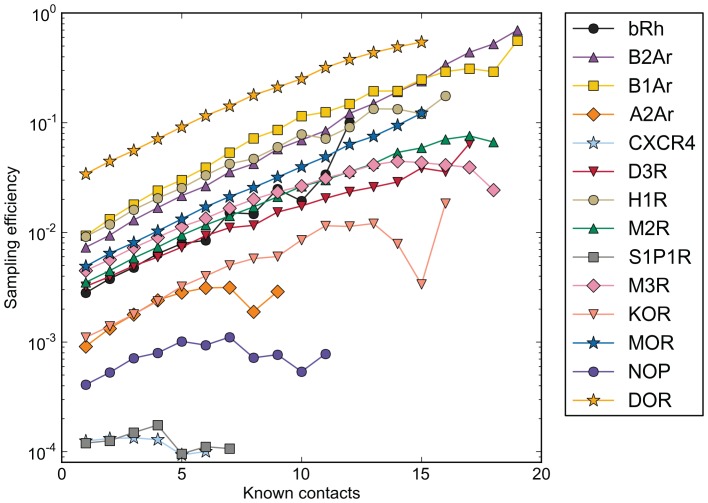
Enriching binding modes with known receptor-ligand contacts increase sampling efficiency of native-like binding modes. For binding modes generated for each receptor, a random number of known contacts from 0 to the greatest possible number of contacts were chosen for 50,000 iterations and the fraction of binding modes sampled within 2.0 Å of the experimental binding mode is given.

Docking into comparative models guided by mutational data is a widespread and largely non-validated method in the literature. Typically, side-chain alterations that heavily affect ligand binding are interpreted as having direct contacts to the ligand. To assess how such information can be used as experimental constraints in our ligand-protein ensembles, we tested to which extent these constraints would allow us to detect the correct binding mode. Enrichment of the correct binding modes was determined through 10,000 iterations of randomly chosen contacts between 0 and total number of all 4.0 Å contacts between the ligand and receptor. When docking ligand conformers into comparative models, the sampling efficiency for native-like binding modes increased on average by one log scale for every 10 known contacts assumed between the binding mode and the receptor (Figure 12). The greatest improvement was seen for receptors where sampling efficiency of the experimental binding mode was already above 0.1%, particularly for DOR, NOP and B1Ar. Little or no improvement in sampling efficiency was observed for those receptor ensembles already sampling less than 0.1% of the experimental binding mode, including A2Ar, B2Ar, S1P1R and CXCR4. Experimental data with higher information density, such as the ionic interactions used for blinded prediction of the binding mode of eticlopride in the dopamine D3 receptor, can be expected to provide a significantly higher improvement in sampling efficiency – in our ensemble, the sampling efficiency was improved by 7 fold by requiring a distance of less than 3.0 Å between the positively charged hydrogen atom on the tertiary amine and the oxygen atoms in the carboxyl acid group of the aspartic acid.

### Concluding Remarks

This study provides an analysis of the sampling performance that can be expected when docking ligands into comparative models of GPCRs. Previous studies of ligand docking into GPCR comparative models have demonstrated that the problem is highly challenging for all but the simplest of cases that require the least sampling of receptor space [3], [4], [6]. This is in agreement with recent docking studies for flexible ligand docking into multiple static structures [61]–[63], which consistently report that while the performance in docking and screening accuracy of a ‘small’ conformational ensemble is superior to that of a single conformer, that performance starts to rapidly decline when the size of the conformational ensemble begins to grow. The study presented here sought to quantify the challenges of docking ensembles of ligand conformers into comparative models through deep and relatively unbiased sampling using full receptor and ligand flexibility.

Comparative models of 14 unique GPCRs were constructed using the other 13 experimental structures as templates. Threading was based on the unbiased alignment between the target and template sequences and loops were constructed *de novo* with a fragment-based loop closure algorithm in Rosetta. When compared to corresponding experimental structures, the most accurate comparative models demonstrated a correlation to Rosetta energy. Top ranked structures with templates within 50% sequence identity were modeled with an average RMSD of 2.2 Å in the transmembrane region, with the best models coming within 1.2 Å RMSD. Extracellular loops with lengths ranging between 5 and 7 residues were modeled with an average RMSD of 2.0 Å, while ECL2 was modeled with an average RMSD of 5.3 Å. The most difficult cases to model were those in which helical regions were unable to align to suitable templates and those cases in which N-terminal residues necessary for ECL2 packing were missing. Despite these challenges, Rosetta was still able to rank more accurate loop conformations than other leading methods.

Using the ligands found in the crystallized GPCR structures, docking was performed in the top ranked comparative models. Docking ensembles of ligand conformers into comparative models sampled the correct ligand binding mode for each of the 14 receptors, but often less than 1% of the time. While the lack of energy gap makes discrimination of the correct binding modes difficult, certain techniques for filtering the models and binding modes demonstrated some success in this study. Using templates with a sequence identity above 50% provides a higher chance for correctly modeling of the ligand binding pocket as also observed in previous studies [6]. In cases where such templates do not exist, using a knowledge-based filter to identify models for which the binding pocket is within the variability that is represented in the experimental structures is beneficial for docking, significantly increasing sampling efficiency in 10 of the 14 cases. Inaccuracies in the minimized structures strongly affected the accuracy in the loop regions, which in turn affected the resulting ligand binding modes. Therefore, it may be best to limit the flexibility introduced by sampling when using a highly homologous template, such as the case for B1Ar and B2Ar.

As demonstrated in this study, clustering can provide improvement over energy for identifying correct binding modes, but only if clusters contain at least 1% or more of the total binding modes. Selection of the correct binding mode from an ensemble of models might be further improved using information from structure activity relationship of active ligands, as proposed by Katritch et al., to select the best performing models from an ensemble [64]. However, this requires knowledge about active ligands which is typically limited for novel protein or receptor targets and the approach is based on the assumption that different ligands share a common binding mode.

In addition, many papers are published under the premise that experimental information such as mutational data can aid in finding the correct ligand binding mode within a large ensemble of models [65]–[69]. Considering the challenges faced in this study, application of mutational data as experimental constraints seems to be an appealing strategy. Sampling efficiency for binding modes within 2.0 Å of the experimental ligand binding mode increased on average by one log scale for every 10 known contacts between the binding mode and the receptor. However, the expected benefit should be evaluated carefully on one or more experimental ligand-receptor complexes to access the true value of such constraints – in particular since indirect effects are known to occur and could blur the identification and the selection of the “correct” binding modes.

Through the use of unbiased sequence alignments and sampling algorithms using the Rosetta software suite, the most challenging scenario for GPCR comparative modeling and ligand docking was explored. As with other studies on comparative modeling and docking, however, there were still minor biases introduced in both aspects of this work which may limit the scope of this approach. Bias in the comparative modeling experiments included the addition of constraints on the disulfide connectivity of the loops based on the experimental structures, which influenced the conformations of ECL2. Bias in the ligand docking experiments included the ligand stereochemistry and protonation state of CXCR4, restricting the conformational search space by centering a sphere of 5Å radius at the center of the experimentally determined binding mode. Also, bias to the experimentally determined structures could have been eliminated with a leave-one-out cross-validation of the knowledge based filter. Despite these biases, the findings of this study identified specific avenues for improvement to approach this challenging problem. Knowledge-based and energy-based filters are able to improve sampling performance over random by 10^3^ fold. Additionally, sampling performance is increased by one order of magnitude for every 10 residues known to contact the ligand. Contacts with high information density, specifically the salt bridge between the oxygen atoms of an aspartic acid in DOR and the positively charged hydrogen atom on the tertiary amine of its ligand, improved sampling efficiency 7 fold. As the number of GPCR experimental structures being solved increases, so does the opportunity to find suitable templates for comparative modeling. With the guidelines suggested by the results from this study, relevant ligand docking studies may be able to generate structural hypotheses to guide experimental designs.

## Supporting Information

Figure S1 **Ligand structures used in this study.** Ligand structures depicted here were crystallized with the G-protein coupled receptors used in this study and were obtained from the Protein Data Bank.
(TIF)

Figure S2 **Structure-based sequence alignment of G-protein coupled receptors.** This sequence alignment of the fourteen GPCRs used in this study was obtained through a structural alignment of the receptors in MUSTANG [28]. Transmembrane regions are highlighted in blue, cysteine residues forming disulfide bonds are highlighted in yellow and residues in contact with their respective ligands are highlighted in purple. Conserved residues representing Ballesteros-Weinstein x.50 are outlined with a black box. The figure was generated using Aline [44].
(TIF)

Figure S3 **Structures of G-protein coupled receptors used in this study.** Experimental structures of the fourteen G-protein coupled receptors used in this study were obtained from the Protein Data Bank. Extracellular loop (ECL) 1 is shown in yellow, ECL2 in purple, and ECL3 in orange.
(TIF)

Figure S4 **Energy plot of relaxed experimental** **structures and comparative models compared with full receptor RMSD.** For each structure, full receptor RMSD is plotted against total Rosetta energy. The experimental structure was minimized in the Rosetta force field without the ligand (in green) and with the ligand (in blue). Comparative models are in grey, with models selected through clustering in orange and models selected by the knowledge-based filter in purple.
(TIF)

Figure S5 **Energy plot of comparative models based on templates of varying sequence identity.** For each comparative model, pocket residue RMSD is plotted against total Rosetta energy. Each point is colored by the template by which the model was built, with color varying from blue to red with increasing sequence identity.
(TIF)

Figure S6 **Energy plot of ECL1 in comparative models.** For each comparative model, ECL1 RMSD is plotted against the Rosetta energy for residues in ECL1.
(TIF)

Figure S7 **Energy plot of ECL2 in comparative models.** For each comparative model, ECL2 RMSD is plotted against the Rosetta energy for residues in ECL2. ECL2 for A2Ar and H1R could not be evaluated because of unresolved structure in this region of the experimental structure in the Protein Data Bank.
(TIF)

Figure S8 **Energy plot of ECL3 in comparative models.** For each comparative model, ECL3 RMSD is plotted against the Rosetta energy for residues in ECL3. ECL3 for KOR could not be evaluated because of unresolved structure in this region of the experimental structure in the Protein Data Bank.
(TIF)

Figure S9 **Structural representations of extracellular loop two from comparative models compared to experimental structures.** For **A)** bRh, **B)** B1Ar, **C)** B2Ar, **D)** A2Ar, **E)** CXCR4, **F)** D3R, **G)** H1R, **H)** S1P1R, **I)** M2R, **J)** M3R, **K)** MOR, **L)** KOR, **M)** NOP and **N)** DOR, the experimental structure is represented in gray, the most accurately sampled model is represented in yellow and the top ranked model is represented in blue. The top ranked model is the lowest energy model of the largest cluster, where clustering is performed on pairwise full receptor C-alpha RMSD over the top ten percent of comparative models by energy.
(TIF)

Figure S10 **RMSD of ligand conformations generated by MOE.** Ligand conformations generated by MOE using the MMFF94x force field and Generalized Born solvation model were compared to the bioactive conformation found in the experimental structure by RMSD to heavy atoms in the ligand. The average RMSD is represented by a black line. The fold decrease in sampling efficiency is calculated by the uniform sampling efficiency within a 2.0 Å radius (USE2.0) for the bioactive ligand conformation divided by the uniform sampling efficiency within a 2.0 Å radius for ligand conformers.
(TIF)

Figure S11 **Interaction energy plot of binding modes from docking into experimental structures and comparative models**. For each structure, ligand heavy-atom RMSD is plotted against Rosetta interaction energy. The bioactive ligand conformation was docked into the static experimental structure (in blue), the energy minimized experimental structure (in orange) and comparative models (in purple). Ligand conformers generated by MOE were docked into comparative models, shown in yellow.
(TIF)

Figure S12 **High sequence identity templates produce models with more accurate binding modes**. Each point represents the average ligand RMSD over all binding modes produced by docking the ligand into target GPCR comparative models built using a particular template. For each target-template pair, percent sequence identity was calculated on the sequence alignment shown in Figure S2. Sequence identity is shown here to correlate with low average ligand heavy-atom RMSD.
(TIF)

Table S1 **Rosetta loop modeling in comparative models with cyclic coordinate descent compared to kinematic loop closure.** Reported is the average RMSD and standard deviation for all comparative models of target receptors, calculated over C-alpha atoms in the loop regions compared to the corresponding experimental structure from the Protein Data Bank. Loop closure with KIC was only performed on a subset of the GPCR dataset.
(DOCX)

File S1 **Protocol capture.** This protocol capture contains the steps necessary to obtain the results presented in the manuscript “Assessment and challenges of ligand docking into comparative models of G-protein coupled receptors”. The input files necessary to carry out the steps outlined in this protocol as well as the output files relating to the results found in the manuscript are provided in the attached folder: File S2. GPCR_model_dock.zip. While the actual protocol was carried on every pairwise combination of GPCRs from Table 1, this protocol capture uses the comparative modeling of bRh onto the template B2Ar as an example for simplification. The Rosetta 3.4 software suite is publically available and the license is free for non-commercial users at http://www.rosettacommons.org/. The supplementary materials are included with Rosetta 3.5 under the directory “rosetta_demos/protocol_capture/2012/GPCR_model_dock”.
(DOCX)

File S2 **Files for protocol capture**. The input files necessary to carry out the steps outlined in the protocol capture in File S1 as well as the output files relating to the results found in the manuscript are provided in this attachment.
(ZIP)

## References

[1] Lagerstrom MC, Schioth HB (2008) Structural diversity of G protein-coupled receptors and significance for drug discovery. Nat Rev Drug Discov 7: 339–357. 18382464 10.1038/nrd2518

[2] Overington JP, Al-Lazikani B, Hopkins AL (2006) How many drug targets are there? Nat Rev Drug Discov 5: 993–996. 17139284 10.1038/nrd2199

[3] Michino M, Abola E, Brooks CL, Dixon JS, Moult J, et al (2009) Community-wide assessment of GPCR structure modelling and ligand docking: GPCR Dock 2008. Nature Reviews Drug Discovery 8: 455–463. 19461661 10.1038/nrd2877 PMC2728591

[4] Kufareva I, Rueda M, Katritch V, Stevens R, Abagyan R (2011) Status of GPCR Modeling and Docking as Reflected by Community-wide GPCR Dock 2010 Assessment. Structure 19: 1108–1126. 21827947 10.1016/j.str.2011.05.012 PMC3154726

[5] Rasmussen S, Choi H, Rosenbaum D, Kobilka T, Thian F, et al (2007) Crystal structure of the human β2 adrenergic G-protein-coupled receptor. Nature 450: 383–387. 17952055 10.1038/nature06325

[6] Beuming T, Sherman W (2012) Current Assessment of Docking into GPCR Crystal Structures and Homology Models: Successes, Challenges, and Guidelines. Journal of Chemical Information and Modeling 52: 3263–3277. 23121495 10.1021/ci300411b

[7] Fleishman S, Baker D (2012) Role of the Biomolecular Energy Gap in Protein Design, Structure, and Evolution. Cell 149: 262–273. 22500796 10.1016/j.cell.2012.03.016

[8] Nygaard R, Zou Y, Dror RO, Mildorf TJ, Arlow DH, et al (2013) The dynamic process of beta(2)-adrenergic receptor activation. Cell 152: 532–542. 23374348 10.1016/j.cell.2013.01.008 PMC3586676

[9] Chen KY, Zhou F, Fryszczyn BG, Barth P (2012) Naturally evolved G protein-coupled receptors adopt metastable conformations. Proc Natl Acad Sci U S A 109: 13284–13289. 22847407 10.1073/pnas.1205512109 PMC3421219

[10] Schwartz TW, Rosenkilde MM (1996) Is there a ‘lock’ for all agonist 'keys' in 7TM receptors? Trends in Pharmacological Sciences 17: 213–216. 8763197 10.1016/0165-6147(96)10017-1

[11] Klco JM, Wiegand CB, Narzinski K, Baranski TJ (2005) Essential role for the second extracellular loop in C5a receptor activation. Nature structural & molecular biology 12: 320–326. 10.1038/nsmb913 15768031

[12] Kaufmann KW, Meiler J (2012) Using RosettaLigand for small molecule docking into comparative models. PLoS ONE 7: e50769. 23239984 10.1371/journal.pone.0050769 PMC3519832

[13] Leaver-Fay A, Tyka M, Lewis SM, Lange OF, Thompson J, et al (2011) ROSETTA3: an object-oriented software suite for the simulation and design of macromolecules. Methods Enzymol 487: 545–574. 21187238 10.1016/B978-0-12-381270-4.00019-6 PMC4083816

[14] Okada T, Sugihara M, Bondar A, Elstner M, Entel P, et al (2004) The retinal conformation and its environment in rhodopsin in light of a new 2.2 A crystal structure. Journal of Molecular Biology 342: 571–583. 15327956 10.1016/j.jmb.2004.07.044

[15] Warne T, Serrano-Vega MJ, Baker JG, Moukhametzianov R, Edwards PC, et al (2008) Structure of a β1-adrenergic G-protein-coupled receptor. Nature 454: 486–491. 18594507 10.1038/nature07101 PMC2923055

[16] Cherezov V, Rosenbaum DM, Hanson MA, Rasmussen SGF, Thian FS, et al (2007) High-Resolution Crystal Structure of an Engineered Human 2-Adrenergic G Protein Coupled Receptor. Science 318: 1258–1265. 17962520 10.1126/science.1150577 PMC2583103

[17] Jaakola V, Griffith MT, Hanson MA, Cherezov V, Chien EY, et al (2008) The 2.6 angstrom crystal structure of a human A2A adenosine receptor bound to an antagonist. Science (New York, NY) 322: 1211–1217. 10.1126/science.1164772 PMC2586971 18832607

[18] Wu B, Chien EY, Mol CD, Fenalti G, Liu W, et al (2010) Structures of the CXCR4 chemokine GPCR with small-molecule and cyclic peptide antagonists. Science (New York, NY) 330: 1066–1071. 10.1126/science.1194396 PMC3074590 20929726

[19] Chien EY, Liu W, Zhao Q, Katritch V, Han GW, et al (2010) Structure of the human dopamine D3 receptor in complex with a D2/D3 selective antagonist. Science (New York, NY) 330: 1091–1095. 10.1126/science.1197410 PMC3058422 21097933

[20] Shimamura T, Shiroishi M, Weyand S, Tsujimoto H, Winter G, et al (2011) Structure of the human histamine H1 receptor complex with doxepin. Nature 475: 65–70. 21697825 10.1038/nature10236 PMC3131495

[21] Hanson MA, Roth CB, Jo E, Griffith MT, Scott FL, et al (2012) Crystal structure of a lipid G protein-coupled receptor. Science (New York, NY) 335: 851–855. 10.1126/science.1215904 PMC3338336 22344443

[22] Haga K, Kruse AC, Asada H, Yurugi-Kobayashi T, Shiroishi M, et al (2012) Structure of the human M2 muscarinic acetylcholine receptor bound to an antagonist. Nature 482: 547–551. 22278061 10.1038/nature10753 PMC3345277

[23] Kruse AC, Hu J, Pan AC, Arlow DH, Rosenbaum DM, et al (2012) Structure and dynamics of the M3 muscarinic acetylcholine receptor. Nature 482: 552–556. 22358844 10.1038/nature10867 PMC3529910

[24] Manglik A, Kruse AC, Kobilka TS, Thian FS, Mathiesen JM, et al (2012) Crystal structure of the µ-opioid receptor bound to a morphinan antagonist. Nature 485: 321–326. 22437502 10.1038/nature10954 PMC3523197

[25] Wu H, Wacker D, Mileni M, Katritch V, Han GW, et al (2012) Structure of the human κ-opioid receptor in complex with JDTic. Nature 485: 327–332. 22437504 10.1038/nature10939 PMC3356457

[26] Thompson AA, Liu W, Chun E, Katritch V, Wu H, et al (2012) Structure of the nociceptin/orphanin FQ receptor in complex with a peptide mimetic. Nature 485: 395–399. 22596163 10.1038/nature11085 PMC3356928

[27] Granier S, Manglik A, Kruse AC, Kobilka TS, Thian FS, et al (2012) Structure of the δ-opioid receptor bound to naltrindole. Nature 485: 400–404. 22596164 10.1038/nature11111 PMC3523198

[28] Konagurthu AS, Whisstock JC, Stuckey PJ, Lesk AM (2006) MUSTANG: a multiple structural alignment algorithm. Proteins 64: 559–574. 16736488 10.1002/prot.20921

[29] Thompson JD, Higgins DG, Gibson TJ (1994) CLUSTAL W: improving the sensitivity of progressive multiple sequence alignment through sequence weighting, position-specific gap penalties and weight matrix choice. Nucleic Acids Research 22: 4673–4680. 7984417 10.1093/nar/22.22.4673 PMC308517

[30] Wang C, Bradley P, Baker D (2007) Protein–Protein Docking with Backbone Flexibility. Journal of Molecular Biology 373: 503–519. 17825317 10.1016/j.jmb.2007.07.050

[31] Canutescu AA, Dunbrack RL (2003) Cyclic coordinate descent: A robotics algorithm for protein loop closure. Protein Science 12: 963–972. 12717019 10.1110/ps.0242703 PMC2323867

[32] Yarov-Yarovoy V, Schonbrun J, Baker D (2006) Multipass membrane protein structure prediction using Rosetta. Proteins 62: 1010–1025. 16372357 10.1002/prot.20817 PMC1479309

[33] Mandell DJ, Coutsias EA, Kortemme T (2009) Sub-angstrom accuracy in protein loop reconstruction by robotics-inspired conformational sampling. Nature methods 6: 551–552. 19644455 10.1038/nmeth0809-551 PMC2847683

[34] Alexander N, Woetzel N, Meiler J (2011) Cluster: A method for clustering biological molecules coupled with visualization in the Pymol Molecular Graphics System. Computational Advances in. 10.1109/ICCABS.2011.5729867 PMC5091839 27818847

[35] Labute P (2010) LowModeMD–implicit low-mode velocity filtering applied to conformational search of macrocycles and protein loops. Journal of Chemical Information and Modeling 50: 792–800. 20429574 10.1021/ci900508k

[36] Perola E, Charifson PS (2004) Conformational Analysis of Drug-Like Molecules Bound to Proteins: An Extensive Study of Ligand Reorganization upon Binding. Journal of medicinal chemistry 47: 2499–2510. 15115393 10.1021/jm030563w

[37] Davis IW, Baker D (2009) RosettaLigand docking with full ligand and receptor flexibility. J Mol Biol 385: 381–392. 19041878 10.1016/j.jmb.2008.11.010

[38] Meiler J, Baker D (2006) ROSETTALIGAND: Protein-small molecule docking with full side-chain flexibility. Proteins: Structure, Function, and Bioinformatics 65: 538–548. 10.1002/prot.21086 16972285

[39] Lemmon G, Meiler J (2012) Rosetta Ligand docking with flexible XML protocols. Methods in molecular biology (Clifton, NJ) 819: 143–155. 10.1007/978-1-61779-465-0_10 PMC3749076 22183535

[40] Fleishman SJ, Leaver-Fay A, Corn JE, Strauch E, Khare SD, et al (2011) RosettaScripts: a scripting language interface to the Rosetta macromolecular modeling suite. PLoS ONE 6: 1–10. 10.1371/journal.pone.0020161 PMC3123292 21731610

[41] Bowie JU (1997) Helix packing angle preferences. Nat Struct Biol 4: 915–917. 9360607 10.1038/nsb1197-915

[42] Rueda M, Katritch V, Raush E, Abagyan R (2010) SimiCon: a web tool for protein-ligand model comparison through calculation of equivalent atomic contacts. Bioinformatics (Oxford, England) 26: 2784–2785. 10.1093/bioinformatics/btq504 PMC2981495 20871105

[43] Hunter JD (2007) Matplotlib: A 2D graphics environment. Computing in Science & Engineering 9: 90–95.

[44] Bond CS, Schüttelkopf AW (2009) ALINE: a WYSIWYG protein-sequence alignment editor for publication-quality alignments. Acta crystallographica Section D, Biological crystallography 65: 510–512. 19390156 10.1107/S0907444909007835

[45] Mobarec JC, Sanchez R, Filizola M (2009) Modern homology modeling of G-protein coupled receptors: which structural template to use? Journal of medicinal chemistry 52: 5207–5216. 19627087 10.1021/jm9005252 PMC2891345

[46] Worth CL, Kreuchwig A, Kleinau G, Krause G (2011) GPCR-SSFE: a comprehensive database of G-protein-coupled receptor template predictions and homology models. BMC Bioinformatics 12: 185. 21605354 10.1186/1471-2105-12-185 PMC3113946

[47] Yohannan S (2004) The evolution of transmembrane helix kinks and the structural diversity of G protein-coupled receptors. Proceedings of the National Academy of Sciences 101: 959–963. 10.1073/pnas.0306077101 PMC327124 14732697

[48] Goldfeld D, Zhu K, Beuming T, Friesner R (2012) Loop prediction for a gpcr homology model: Algorithms and resultsLoop prediction for a GPCR homology model. Proteins: 1–15. 10.1002/prot.24178 22965891

[49] Goldfeld DA, Zhu K, Beuming T, Friesner RA (2011) Successful prediction of the intra- and extracellular loops of four G-protein-coupled receptors. Proceedings of the National Academy of Sciences of the United States of America 108: 8275–8280. 21536915 10.1073/pnas.1016951108 PMC3100992

[50] Fiser A, Do RK, Sali A (2000) Modeling of loops in protein structures. Protein science : a publication of the Protein Society 9: 1753–1773. 11045621 10.1110/ps.9.9.1753 PMC2144714

[51] Nikiforovich GV, Taylor CM, Marshall GR, Baranski TJ (2010) Modeling the possible conformations of the extracellular loops in G-protein-coupled receptors. Proteins: Structure, Function, and Bioinformatics 78: 271–285. 10.1002/prot.22537 PMC2795062 19731375

[52] Groban ES, Narayanan A, Jacobson MP (2006) Conformational changes in protein loops and helices induced by post-translational phosphorylation. PLoS Comput Biol 2: e32. 16628247 10.1371/journal.pcbi.0020032 PMC1440919

[53] Cozzini P, Kellogg GE, Spyrakis F, Abraham DJ, Costantino G, et al (2008) Target flexibility: an emerging consideration in drug discovery and design. J Med Chem 51: 6237–6255. 18785728 10.1021/jm800562d PMC2701403

[54] Nicklaus MC, Wang S, Driscoll JS, Milne GW (1995) Conformational changes of small molecules binding to proteins. Bioorganic & medicinal chemistry 3: 411–428. 8581425 10.1016/0968-0896(95)00031-b

[55] Boström J, Norrby PO, Liljefors T (1998) Conformational energy penalties of protein-bound ligands. Journal of Computer-Aided Molecular Design 12: 383–396. 9777496 10.1023/a:1008007507641

[56] Kumar A, Voet A, Zhang KY (2012) Fragment based drug design: from experimental to computational approaches. Curr Med Chem 19: 5128–5147. 22934764 10.2174/092986712803530467

[57] Kumar A, Zhang KY (2012) Computational fragment-based screening using RosettaLigand: the SAMPL3 challenge. J Comput Aided Mol Des 26: 603–616. 22246345 10.1007/s10822-011-9523-0

[58] Mortier J, Rakers C, Frederick R, Wolber G (2012) Computational tools for in silico fragment-based drug design. Curr Top Med Chem 12: 1935–1943. 23116473 10.2174/156802612804547371

[59] Friesner RA, Banks JL, Murphy RB, Halgren TA, Klicic JJ, et al (2004) Glide: a new approach for rapid, accurate docking and scoring. 1. Method and assessment of docking accuracy. Journal of medicinal chemistry 47: 1739–1749. 15027865 10.1021/jm0306430

[60] Sherman W, Day T, Jacobson MP, Friesner RA, Farid R (2006) Novel procedure for modeling ligand/receptor induced fit effects. Journal of medicinal chemistry 49: 534–553. 16420040 10.1021/jm050540c

[61] Bottegoni G, Kufareva I, Totrov M, Abagyan R (2009) Four-dimensional docking: a fast and accurate account of discrete receptor flexibility in ligand docking. Journal of medicinal chemistry 52: 397–406. 19090659 10.1021/jm8009958 PMC2662720

[62] Barril X, Morley SD (2005) Unveiling the full potential of flexible receptor docking using multiple crystallographic structures. Journal of medicinal chemistry 48: 4432–4443. 15974595 10.1021/jm048972v

[63] Rueda M, Bottegoni G, Abagyan R (2010) Recipes for the selection of experimental protein conformations for virtual screening. Journal of Chemical Information and Modeling 50: 186–193. 20000587 10.1021/ci9003943 PMC2811216

[64] Katritch V, Rueda M, Abagyan R (2012) Ligand-guided receptor optimization. Methods in molecular biology (Clifton, NJ) 857: 189–205. 10.1007/978-1-61779-588-6_8 22323222

[65] Jacobson KA, Jayasekara MP, Costanzi S (2012) Molecular Structure of P2Y Receptors: Mutagenesis, Modeling, and Chemical Probes. Wiley Interdiscip Rev Membr Transp Signal 1. 10.1002/wmts.68 PMC3547624 23336097

[66] Hoyer I, Haas AK, Kreuchwig A, Schulein R, Krause G (2013) Molecular sampling of the allosteric binding pocket of the TSH receptor provides discriminative pharmacophores for antagonist and agonists. Biochem Soc Trans 41: 213–217. 23356285 10.1042/BST20120319 PMC3561627

[67] Wang CD, Buck MA, Fraser CM (1991) Site-directed mutagenesis of alpha 2A-adrenergic receptors: identification of amino acids involved in ligand binding and receptor activation by agonists. Mol Pharmacol 40: 168–179. 1678850

[68] Parry JJ, Chen R, Andrews R, Lears KA, Rogers BE (2012) Identification of critical residues involved in ligand binding and G protein signaling in human somatostatin receptor subtype 2. Endocrinology 153: 2747–2755. 22495673 10.1210/en.2011-1662 PMC3359596

[69] Gelis L, Wolf S, Hatt H, Neuhaus EM, Gerwert K (2011) Prediction of a Ligand-binding Niche within a Human Olfactory Receptor by Combining Site-directed Mutagenesis with Dynamic Homology Modeling. Angew Chem Int Ed Engl. 10.1002/anie.201103980 22144177

