# Supplementary figures and images for: Assessment and Challenges of Ligand Docking into Comparative Models of G-Protein Coupled Receptors
Source: PLoS One. 2013 Jul 2;8(7):e67302. doi: 10.1371/journal.pone.0067302 (PMC3699586; doi:10.1371/journal.pone.0067302)

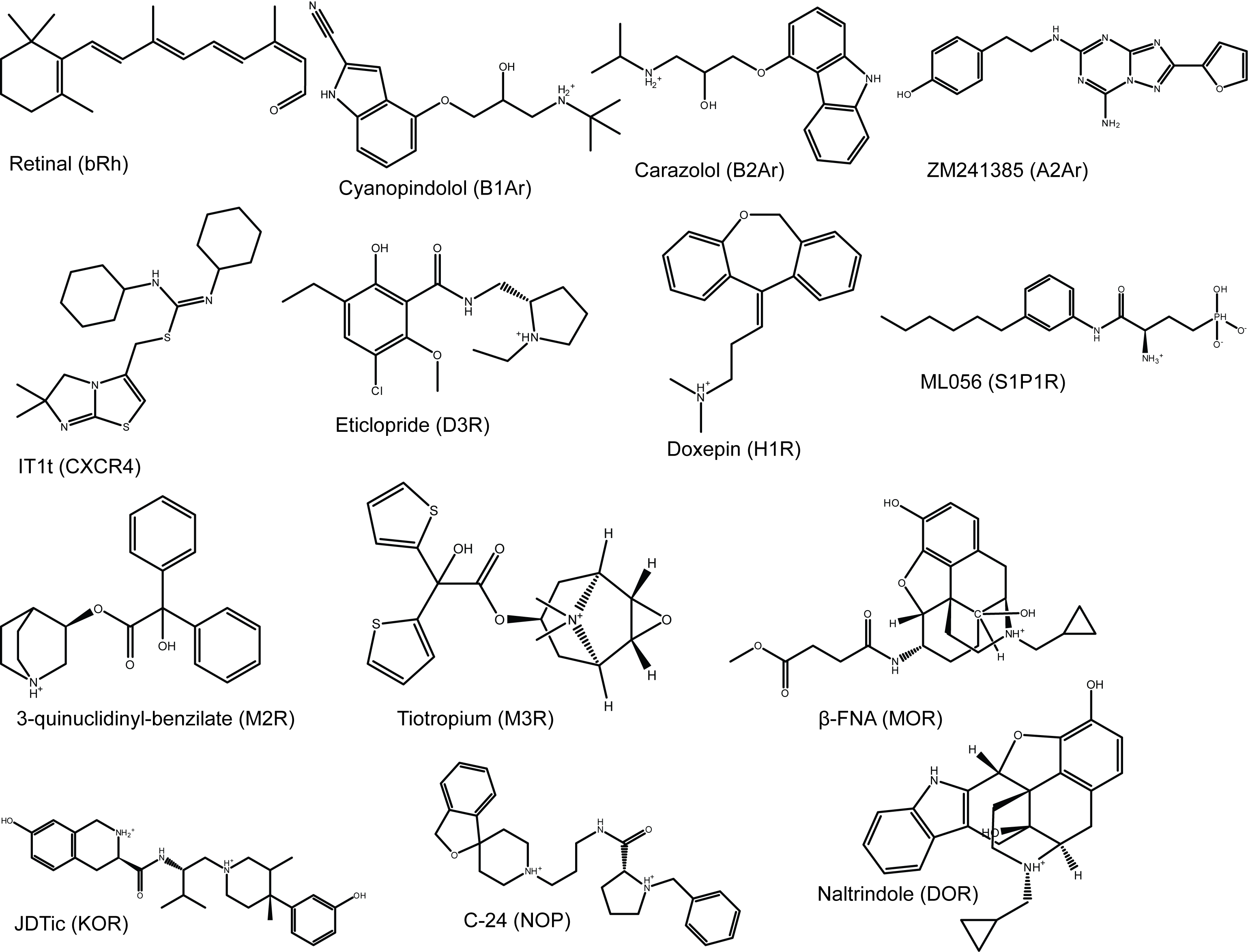

Supplement: Figure S1 — Ligand structures used in this study. Ligand structures depicted here were crystallized with the G-protein coupled receptors used in this study and were obtained from the Protein Data Bank. (TIF) [file pone.0067302.s001.tif]

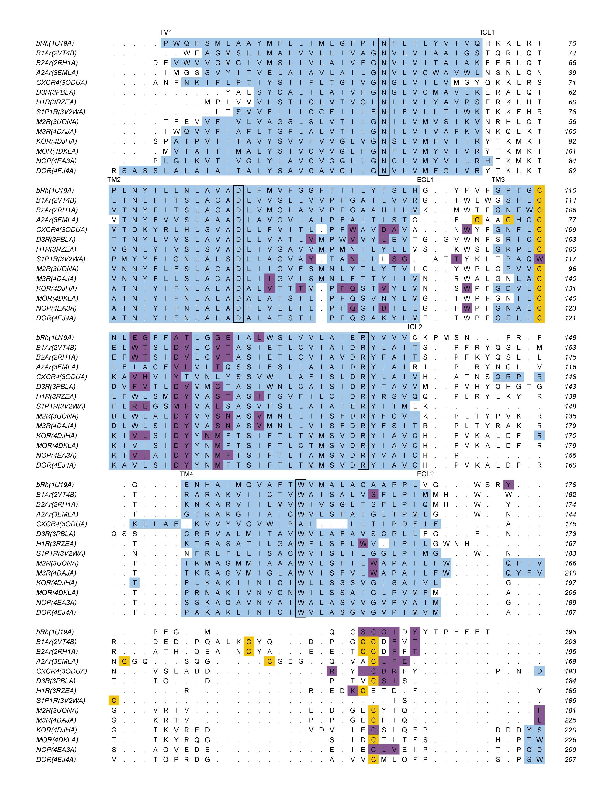

Supplement: Figure S2 — Structure-based sequence alignment of G-protein coupled receptors. This sequence alignment of the fourteen GPCRs used in this study was obtained through a structural alignment of the receptors in MUSTANG [28]. Transmembrane regions are highlighted in blue, cysteine residues forming disulfide bonds are highlighted in yellow and residues in contact with their respective ligands are highlighted in purple. Conserved residues representing Ballesteros-Weinstein x.50 are outlined with a black box. The figure was generated using Aline [44]. (TIF) [file pone.0067302.s002.tif]

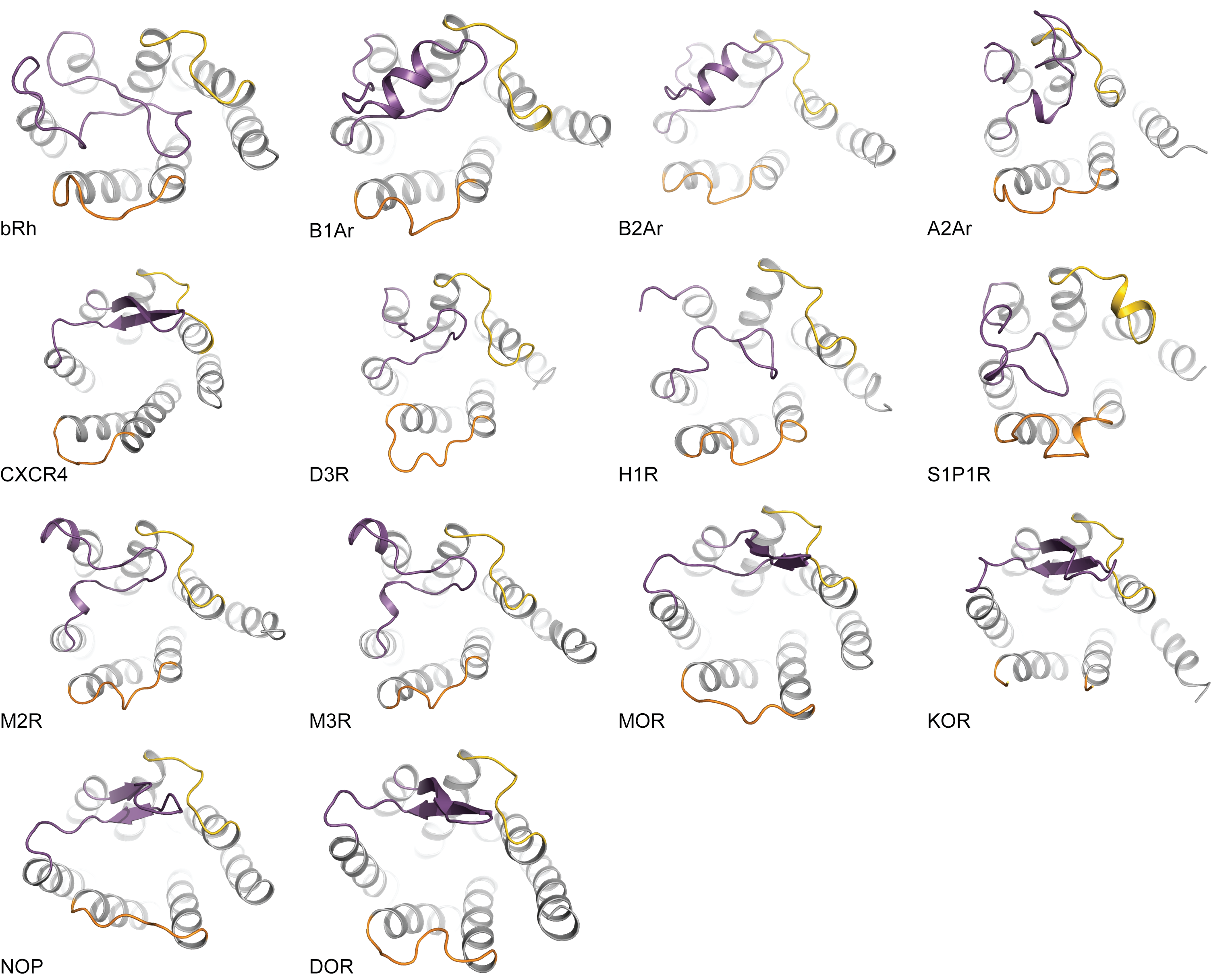

Supplement: Figure S3 — Structures of G-protein coupled receptors used in this study. Experimental structures of the fourteen G-protein coupled receptors used in this study were obtained from the Protein Data Bank. Extracellular loop (ECL) 1 is shown in yellow, ECL2 in purple, and ECL3 in orange. (TIF) [file pone.0067302.s003.tif]

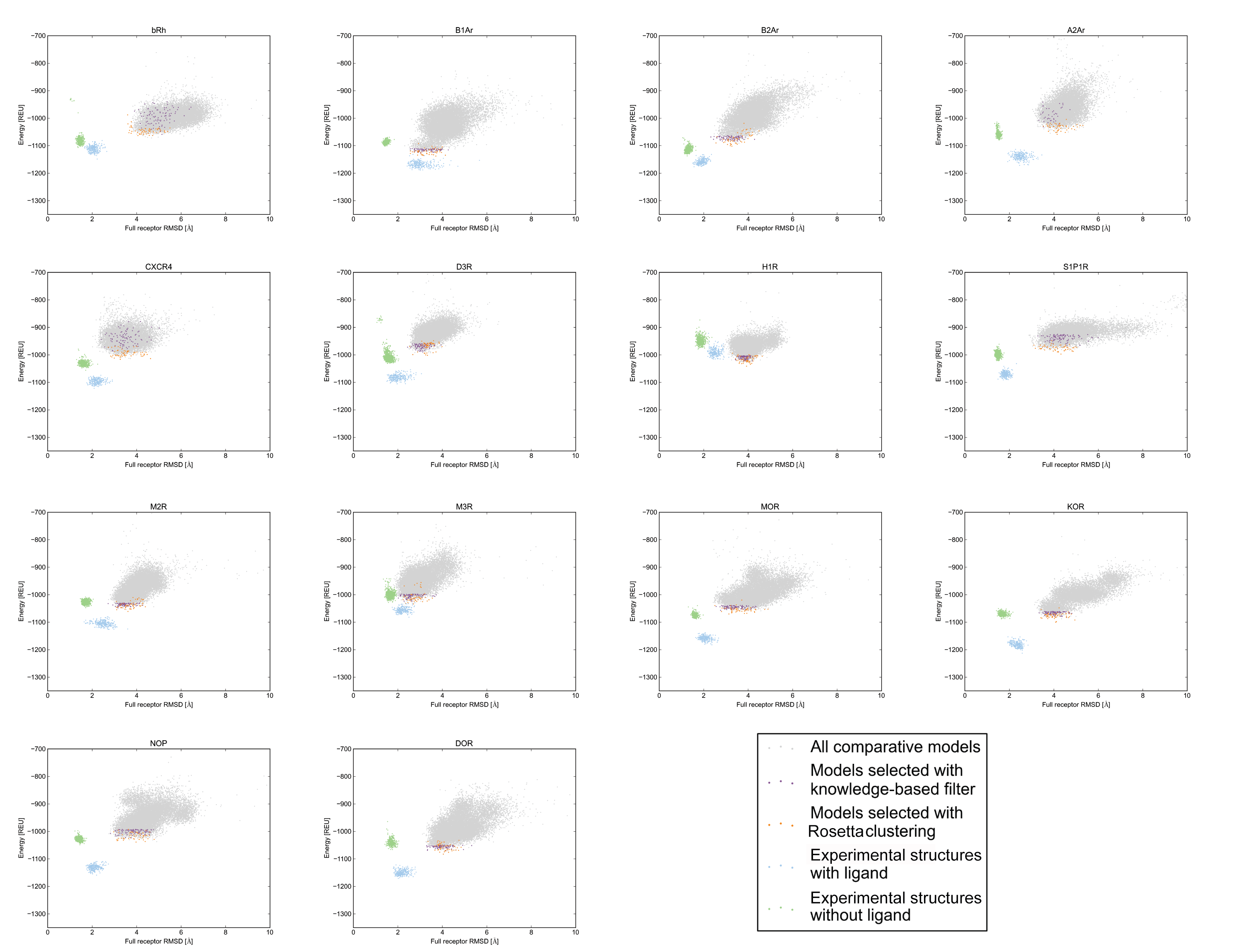

Supplement: Figure S4 — Energy plot of relaxed experimental structures and comparative models compared with full receptor RMSD. For each structure, full receptor RMSD is plotted against total Rosetta energy. The experimental structure was minimized in the Rosetta force field without the ligand (in green) and with the ligand (in blue). Comparative models are in grey, with models selected through clustering in orange and models selected by the knowledge-based filter in purple. (TIF) [file pone.0067302.s004.tif]

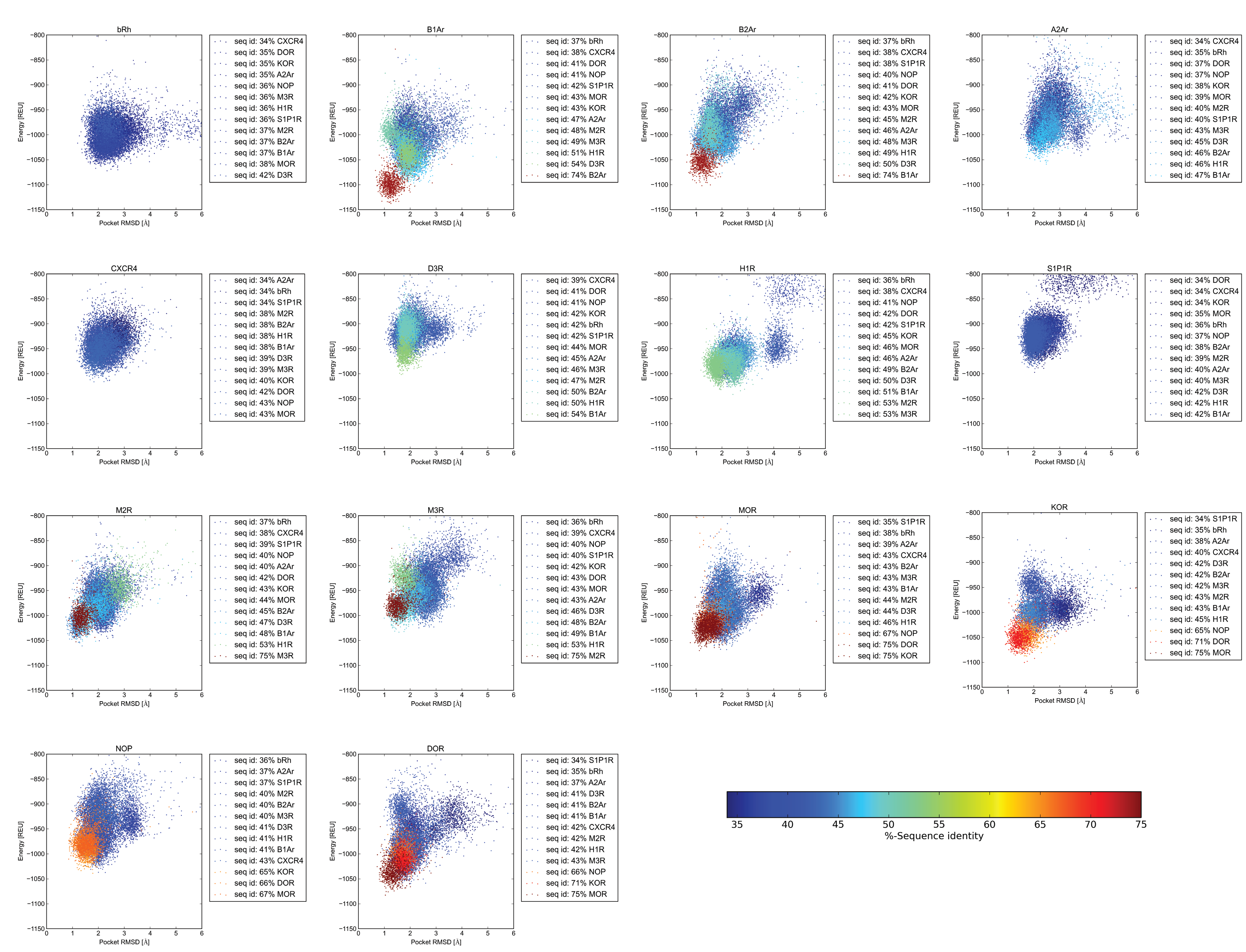

Supplement: Figure S5 — Energy plot of comparative models based on templates of varying sequence identity. For each comparative model, pocket residue RMSD is plotted against total Rosetta energy. Each point is colored by the template by which the model was built, with color varying from blue to red with increasing sequence identity. (TIF) [file pone.0067302.s005.tif]

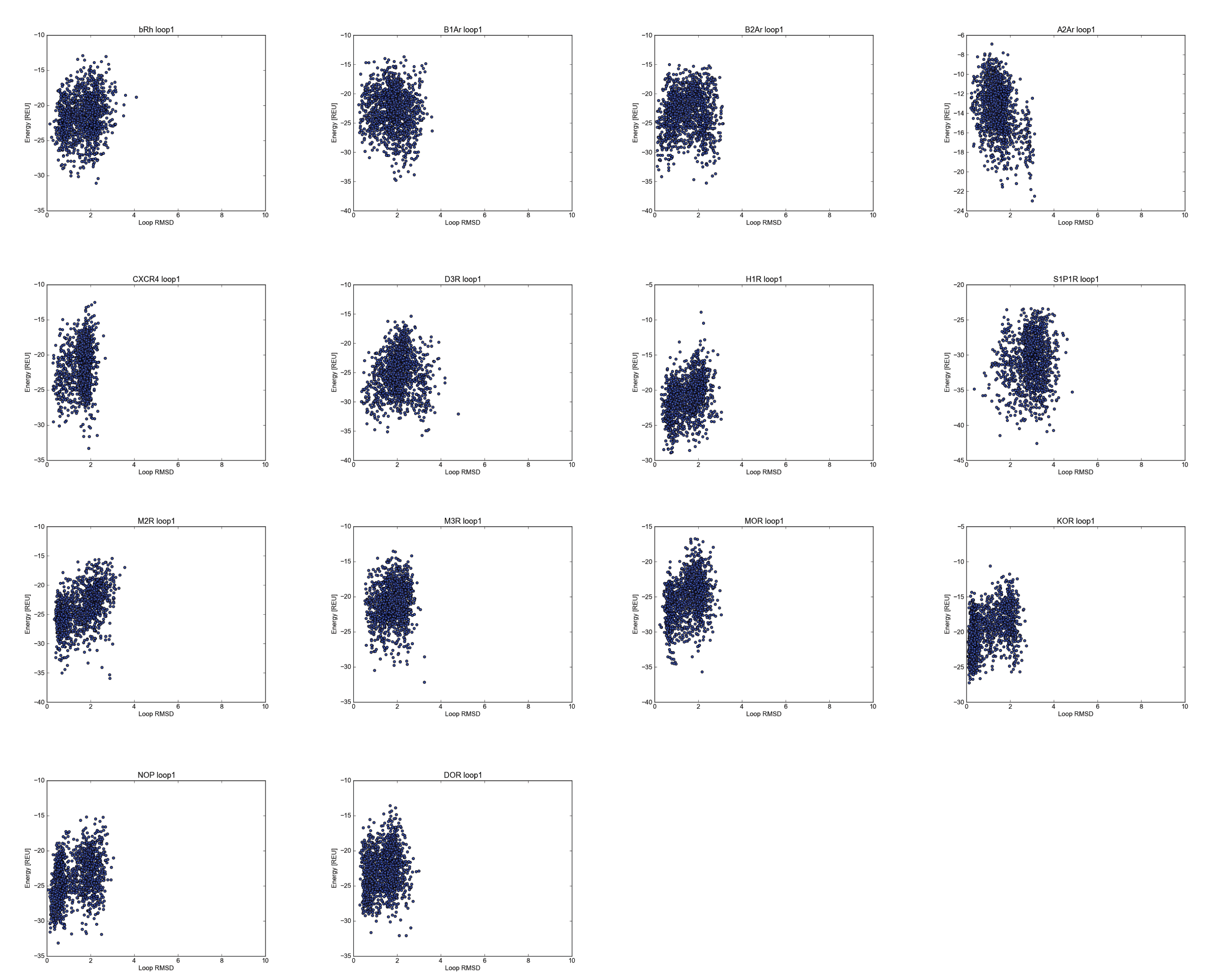

Supplement: Figure S6 — Energy plot of ECL1 in comparative models. For each comparative model, ECL1 RMSD is plotted against the Rosetta energy for residues in ECL1. (TIF) [file pone.0067302.s006.tif]

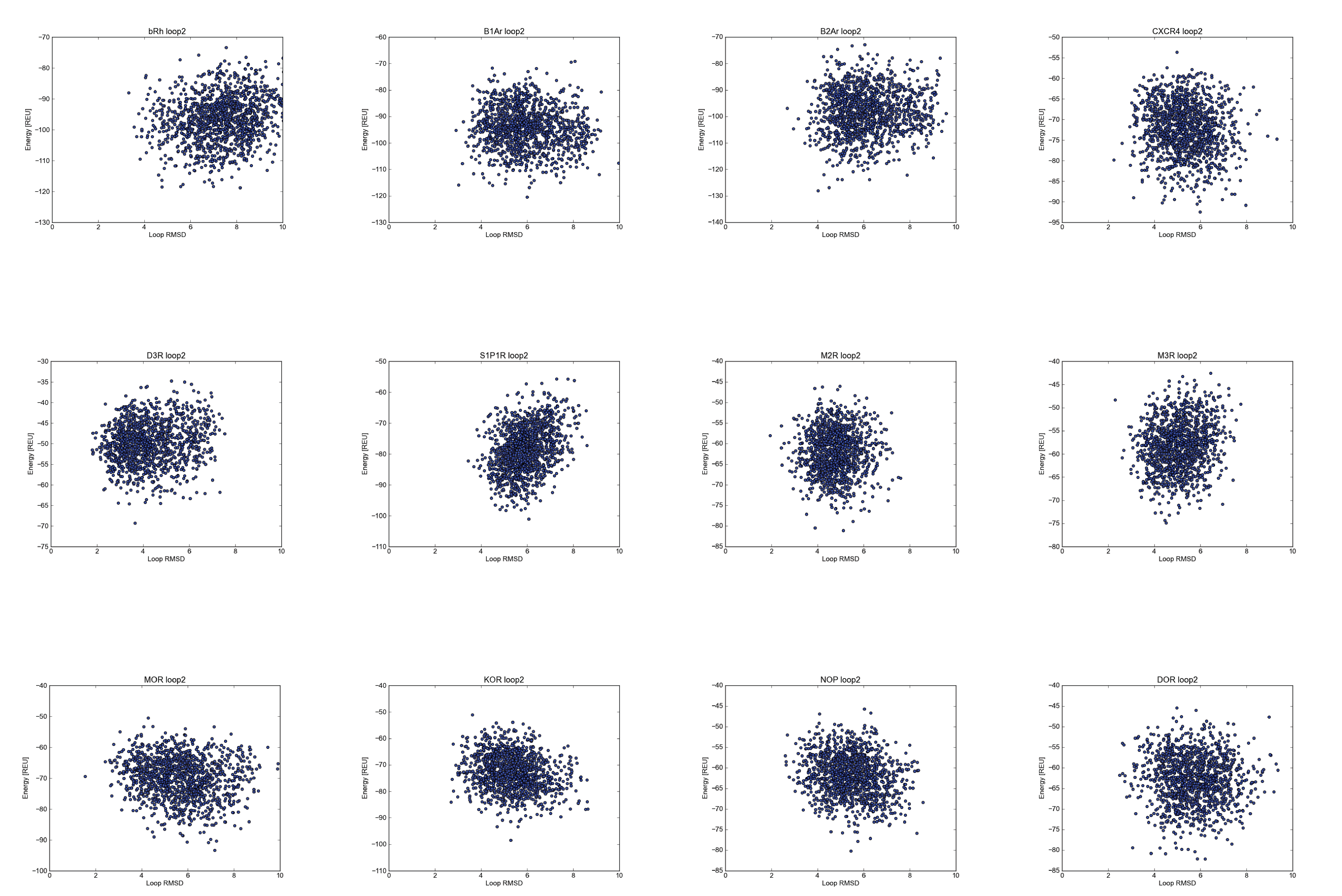

Supplement: Figure S7 — Energy plot of ECL2 in comparative models. For each comparative model, ECL2 RMSD is plotted against the Rosetta energy for residues in ECL2. ECL2 for A2Ar and H1R could not be evaluated because of unresolved structure in this region of the experimental structure in the Protein Data Bank. (TIF) [file pone.0067302.s007.tif]

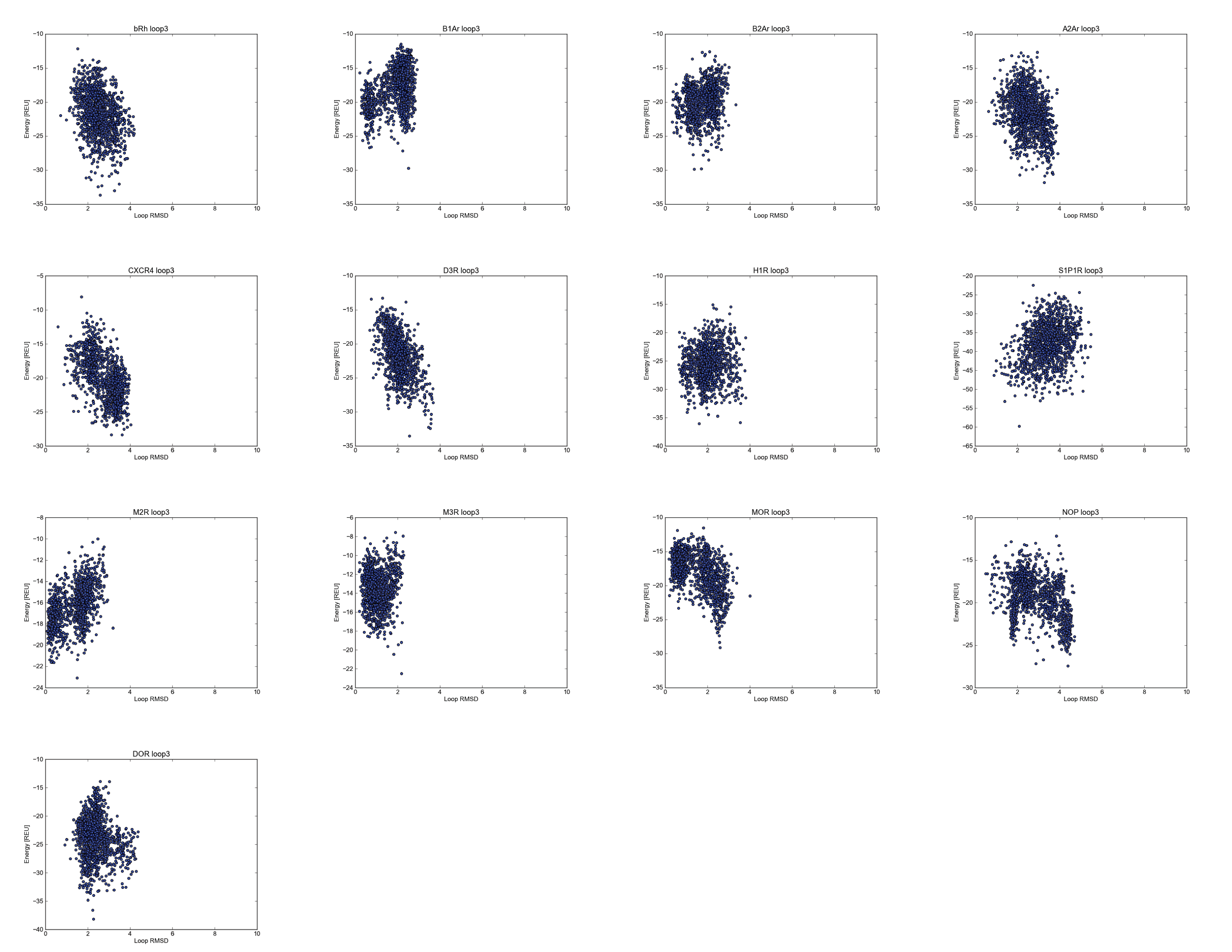

Supplement: Figure S8 — Energy plot of ECL3 in comparative models. For each comparative model, ECL3 RMSD is plotted against the Rosetta energy for residues in ECL3. ECL3 for KOR could not be evaluated because of unresolved structure in this region of the experimental structure in the Protein Data Bank. (TIF) [file pone.0067302.s008.tif]

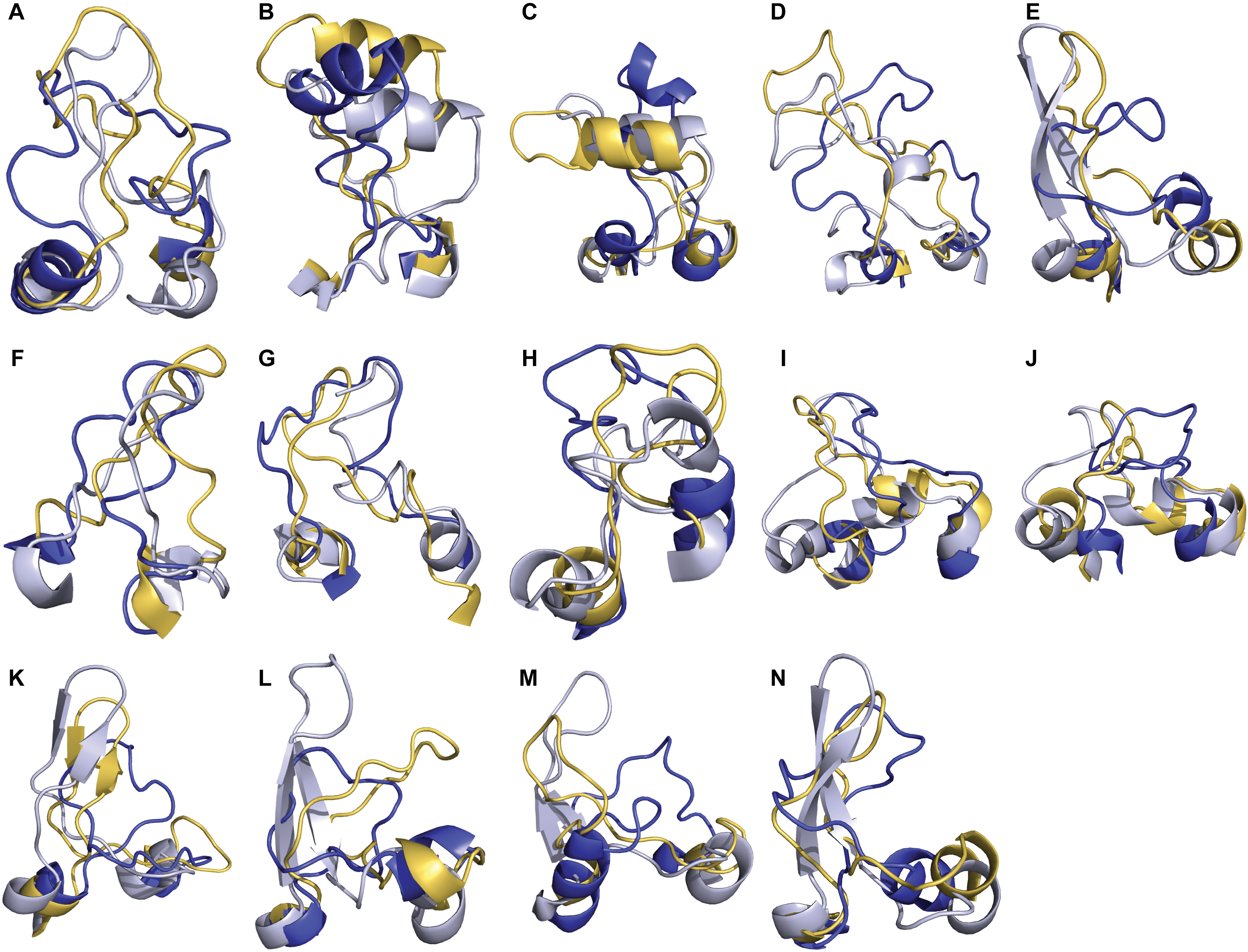

Supplement: Figure S9 — Structural representations of extracellular loop two from comparative models compared to experimental structures. For A) bRh, B) B1Ar, C) B2Ar, D) A2Ar, E) CXCR4, F) D3R, G) H1R, H) S1P1R, I) M2R, J) M3R, K) MOR, L) KOR, M) NOP and N) DOR, the experimental structure is represented in gray, the most accurately sampled model is represented in yellow and the top ranked model is represented in blue. The top ranked model is the lowest energy model of the largest cluster, where clustering is performed on pairwise full receptor C-alpha RMSD over the top ten percent of comparative models by energy. (TIF) [file pone.0067302.s009.tif]

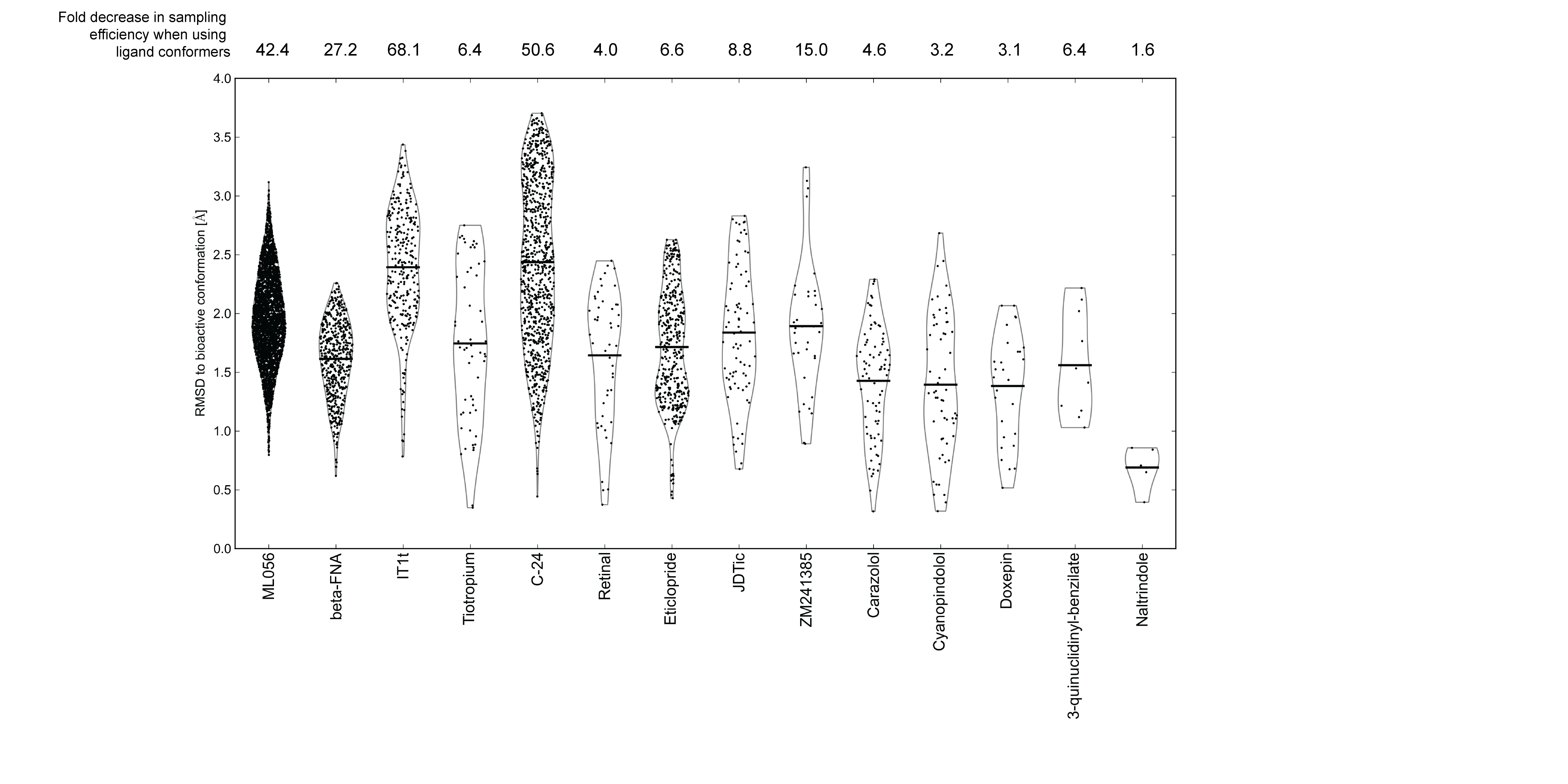

Supplement: Figure S10 — RMSD of ligand conformations generated by MOE. Ligand conformations generated by MOE using the MMFF94x force field and Generalized Born solvation model were compared to the bioactive conformation found in the experimental structure by RMSD to heavy atoms in the ligand. The average RMSD is represented by a black line. The fold decrease in sampling efficiency is calculated by the uniform sampling efficiency within a 2.0 Å radius (USE2.0) for the bioactive ligand conformation divided by the uniform sampling efficiency within a 2.0 Å radius for ligand conformers. (TIF) [file pone.0067302.s010.tif]

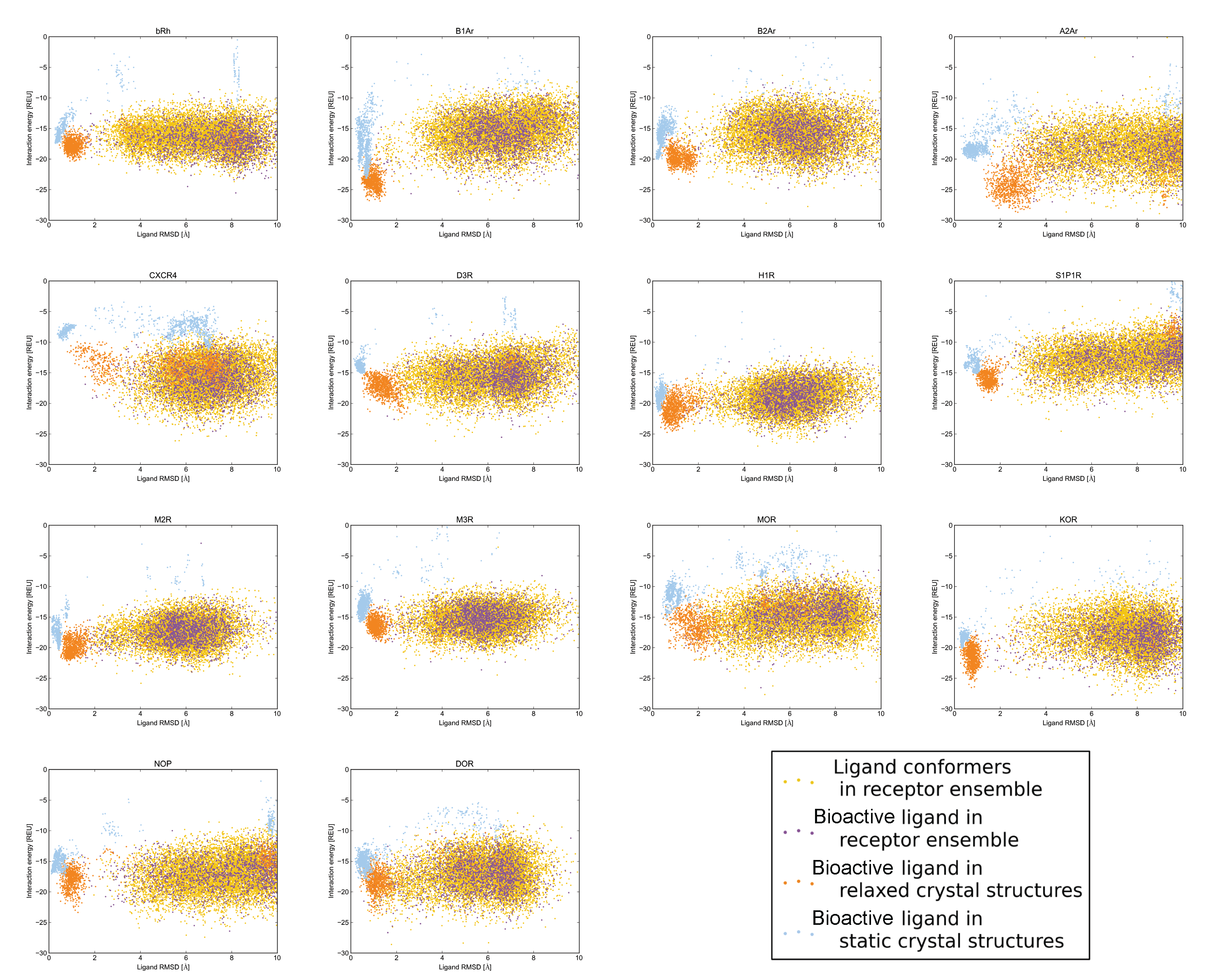

Supplement: Figure S11 — Interaction energy plot of binding modes from docking into experimental structures and comparative models. For each structure, ligand heavy-atom RMSD is plotted against Rosetta interaction energy. The bioactive ligand conformation was docked into the static experimental structure (in blue), the energy minimized experimental structure (in orange) and comparative models (in purple). Ligand conformers generated by MOE were docked into comparative models, shown in yellow. (TIF) [file pone.0067302.s011.tif]

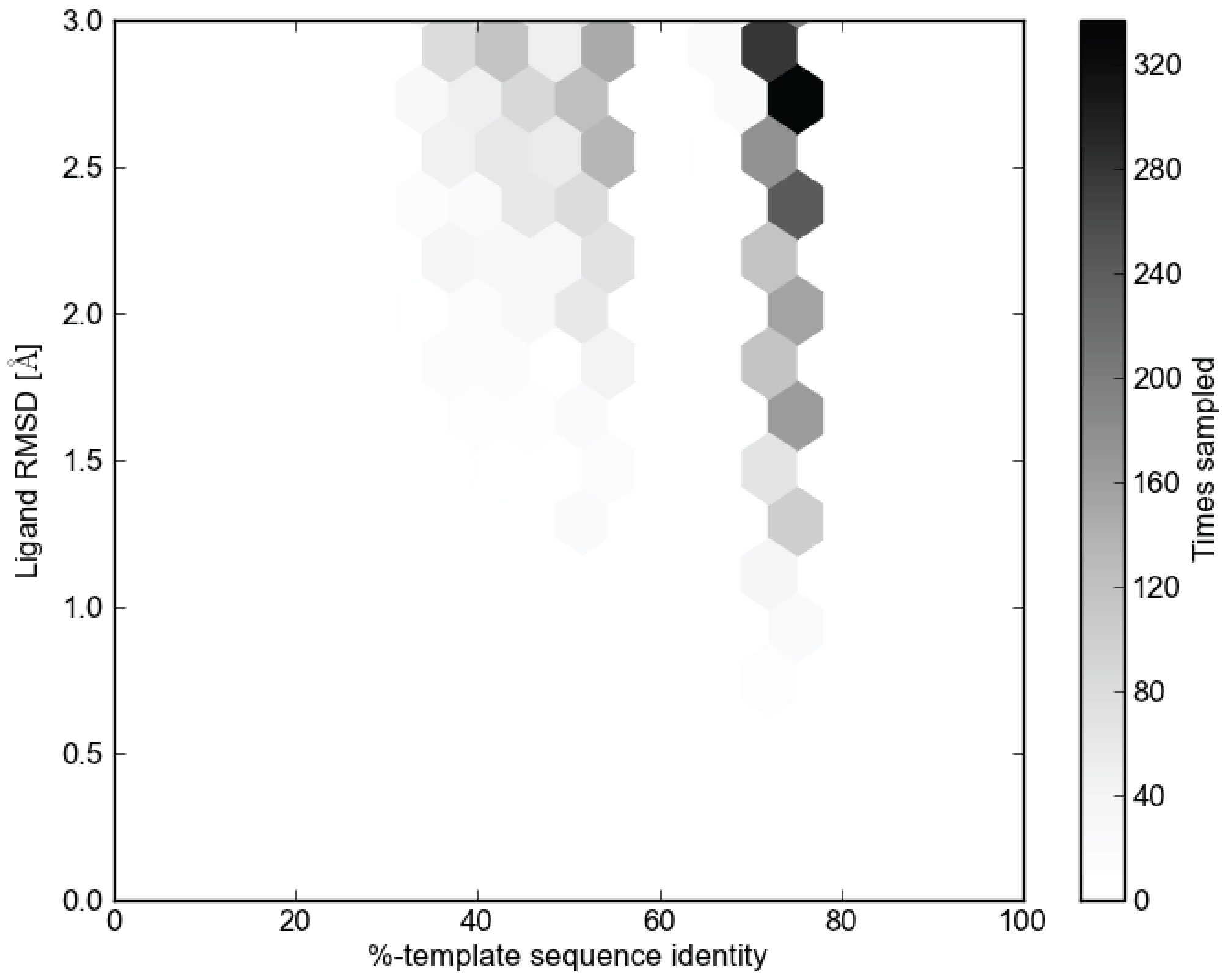

Supplement: Figure S12 — High sequence identity templates produce models with more accurate binding modes. Each point represents the average ligand RMSD over all binding modes produced by docking the ligand into target GPCR comparative models built using a particular template. For each target-template pair, percent sequence identity was calculated on the sequence alignment shown in Figure S2. Sequence identity is shown here to correlate with low average ligand heavy-atom RMSD. (TIF) [file pone.0067302.s012.tif]
