# Supplementary material for: Assessment and Challenges of Ligand Docking into Comparative Models of G-Protein Coupled Receptors
Source: PLoS One. 2013 Jul 2;8(7):e67302. doi: 10.1371/journal.pone.0067302 (PMC3699586; doi:10.1371/journal.pone.0067302)
Supplement: Table S1 — Rosetta loop modeling in comparative models with cyclic coordinate descent compared to kinematic loop closure. Reported is the average RMSD and standard deviation for all comparative models of target receptors, calculated over C-alpha atoms in the loop regions compared to the corresponding experimental structure from the Protein Data Bank. Loop closure with KIC was only performed on a subset of the GPCR dataset. (DOCX) [file pone.0067302.s013.docx]

**Table S1: Rosetta loop modeling in comparative models with cyclic coordinate descent compared to kinematic loop closure.** Reported is the average RMSD and standard deviation for all comparative models of target receptors, calculated over C-alpha atoms in the loop regions compared to the corresponding experimental structure from the Protein Data Bank. Loop closure with KIC was only performed on a subset of the GPCR dataset.

|  | Extracellular Loop 1 RMSD (Å) | | Extracellular Loop 2  RMSD (Å) | | Extracellular Loop 3  RMSD (Å) | | Full Receptor  RMSD (Å) | |
| --- | --- | --- | --- | --- | --- | --- | --- | --- |
|  | CCD | KIC | CCD | KIC | CCD | KIC | CCD | KIC |
| bRh | 1.7±0.7* | 2.0±0.8 | 7.3±1.4 | 7.3±4.2 | 2.6±0.6 | 2.6±0.8 | 4.7±0.6* | 10.6±8.5 |
| B1Ar | 1.8±0.6* | 2.5±0.8 | 6.0±1.2 | 5.7±3.3 | 1.9±0.7* | 2.1±0.5 | 3.6±0.6* | 4.3±3.4 |
| B2Ar | 1.5±0.7* | 3.2±1.2 | 6.2±1.3* | 6.7±3.8 | 1.7±0.5* | 2.1±0.6 | 3.7±0.5* | 4.9±3.4 |
| A2Ar | 1.4±0.5* | 2.2±1.2 | n.d.^a^ | n.d.^a^ | 2.5±0.6* | 2.7±1.4 | 3.4±0.5* | 8.5±5.9 |
| CXCR4 | 1.6±0.4* | 2.5±1.1 | 5.3±1.0* | 6.2±3.3 | 2.8±0.7* | 4.5±3.0 | 4.5±0.4* | 9.3±8.7 |
| D3R | 2.1±0.6* | 2.3±0.9 | 4.3±1.2* | 5.3±3.0 | 2.1±0.5* | 2.9±1.8 | 2.9±0.4* | 4.0±3.1 |
| H1R | 1.5±0.6 | n.d. | n.d.^a^ | n.d.^a^ | 2.0±0.6 | n.d. | 2.5±0.6 | n.d. |
| S1P1R | 2.9±0.6 | n.d. | 5.9±0.8 | n.d. | 3.4±0.8 | n.d. | 4.2±0.9 | n.d. |
| M2R | 1.6±0.8 | n.d. | 4.8±0.8 | n.d. | 1.4±0.7 | n.d. | 2.6±0.4 | n.d. |
| M3R | 1.8±0.5 | n.d. | 5.1±0.9 | n.d. | 1.1±0.4 | n.d. | 2.8±0.4 | n.d. |
| MOR | 1.5±0.6 | n.d. | 5.6±1.4 | n.d. | 1.6±0.8 | n.d. | 3.2±0.9 | n.d. |
| KOR | 1.0±0.8 | n.d. | 5.3±1.0 | n.d. | n.d.^a^ | n.d. | 3.5±0.4 | n.d. |
| NOP | 1.3±0.7 | n.d. | 5.5±1.1 | n.d. | 2.9±0.9 | n.d. | 3.1±0.6 | n.d. |
| DOR | 1.4±0.6 | n.d. | 5.7±1.1 | n.d. | 2.4±0.6 | n.d. | 3.2±0.5 | n.d. |

n.d. denotes not determined
* Indicated significant improvement using the CCD method over KIC for the given category, evaluated with the unpaired t-test, p < 0.05
^a^ Could not be evaluated because of unresolved structure in this region of the experimental structure in the Protein Data Bank.
