# Supplementary material for: Assessment and Challenges of Ligand Docking into Comparative Models of G-Protein Coupled Receptors
Source: PLoS One. 2013 Jul 2;8(7):e67302. doi: 10.1371/journal.pone.0067302 (PMC3699586; doi:10.1371/journal.pone.0067302)
Supplement: File S1 — Protocol capture. This protocol capture contains the steps necessary to obtain the results presented in the manuscript “Assessment and challenges of ligand docking into comparative models of G-protein coupled receptors”. The input files necessary to carry out the steps outlined in this protocol as well as the output files relating to the results found in the manuscript are provided in the attached folder: File S2. GPCR_model_dock.zip. While the actual protocol was carried on every pairwise combination of GPCRs from Table 1, this protocol capture uses the comparative modeling of bRh onto the template B2Ar as an example for simplification. The Rosetta 3.4 software suite is publically available and the license is free for non-commercial users at http://www.rosettacommons.org/. The supplementary materials are included with Rosetta 3.5 under the directory “rosetta_demos/protocol_capture/2012/GPCR_model_dock”. (DOCX) [file pone.0067302.s014.docx]

**File S1. Protocol capture**

This protocol capture contains the steps necessary to obtain the results presented in the manuscript “Assessment and challenges of ligand docking into comparative models of G-protein coupled receptors”. The input files necessary to carry out the steps outlined in this protocol as well as the output files relating to the results found in the manuscript are provided in the attached folder: **File S2. *GPCR_model_dock.zip***. While the actual protocol was carried on every pairwise combination of GPCRs from Table 1, this protocol capture uses the comparative modeling of bRh onto the template B2Ar as an example for simplification. The Rosetta 3.4 software suite is publically available and the license is free for non-commercial users at <http://www.rosettacommons.org/>. The supplementary materials are included with Rosetta 3.5 under the directory “rosetta_demos/protocol_capture/2012/GPCR_model_dock”.

**1. Structural alignment of GPCR templates**

| **Step** | **Text** | **Commands** | **Comment** |
| --- | --- | --- | --- |
| **1A.** Prepare GPCR crystal structures from the Protein Data Bank. | For this study, the lowest resolution crystal structure for each unique GPCR in the Protein Data Bank (PDB) at the time of writing was chosen for comparative modeling and ligand docking, as shown in Table 1 and Figure S1. | **Obtain PDB files:**  Download GPCR crystal structures from the Protein Data Bank at <http://www.rcsb.org>.  **Clean PDB files:**  Clean PDB files using the following script, written here for use with B2Ar:  rosetta_tools/protein_tools/scripts/clean_pdb.py 2RH1 A > 2rh1A_clean.pdb  Remove lines in the PDB file representing the N-terminal, C-terminal and T4-lysozyme regions. | **Input:**  GPCR crystal structure PDB files from the Protein Data Bank at <http://www.rcsb.org>.  **Output:**   1u19A_clean.pdb 2vt4A_clean.pdb 2rh1A_clean.pdb 3emlA_clean.pdb 3oduA_clean.pdb 3pblA_clean.pdb 3rzeA_clean.pdb 3v2wA_clean.pdb 3uonA_clean.pdb 4dajA_clean.pdb 4dklA_clean.pdb 4djhA_clean.pdb 4ea3A_clean.pdb 4ej4A_clean.pdb |
| **1B.** Perform a structural alignment of GPCRs using crystal structures from the Protein Data Bank. | A structural alignment was performed with all 14 GPCR templates using MUSTANG (Konagurthu et al., 2006), as seen in Fig S1. | mustang -p . -i 1u19A_clean.pdb 2vt4A_clean.pdb 2rh1A_clean.pdb 3emlA_clean.pdb 3oduA_clean.pdb 3pblA_clean.pdb 3rzeA_clean.pdb 3v2wA_clean.pdb 3uonA_clean.pdb 4dajA_clean.pdb 4dklA_clean.pdb 4djhA_clean.pdb 4ea3A_clean.pdb 4ej4A_clean.pdb -o all_gpcrs -F fasta -D 2.5 -s ON | **Input:**  1u19A_clean.pdb 2vt4A_clean.pdb 2rh1A_clean.pdb 3emlA_clean.pdb 3oduA_clean.pdb 3pblA_clean.pdb 3rzeA_clean.pdb 3v2wA_clean.pdb 3uonA_clean.pdb 4dajA_clean.pdb 4dklA_clean.pdb 4djhA_clean.pdb 4ea3A_clean.pdb 4ej4A_clean.pdb  **Output:**   all_gpcrs.fasta |

**2. Sequence alignment of the target GPCR to template sequences**

| **Step** | **Text** | **Commands** | **Comment** |
| --- | --- | --- | --- |
| **2A.** Obtain sequence of the target GPCR. |  | Save sequence output from clean_pdb.py into a FASTA file called 1u19A.fasta. | **Input:**  1u19A_clean.pdb  **Output:**   1u19A.fasta |
| **2B.** Sequence alignment of the target GPCR [bRh] to templates [B2Ar]. | The sequence of the target GPCR was then aligned with the profile of structurally aligned templates using CLUSTALW (Thompson et al., 1994). | Input target sequence 1u19A.fasta and profile alignment all_gpcrs.fasta to <http://mobyle.pasteur.fr/cgi-bin/portal.py#forms::clustalO-profile>.  Default settings were used. | **Input:**  1u19A.fasta, all_gpcrs.fasta  **Output:**   1u19A.aln |

**3. Thread target sequence onto template backbone coordinates**

| **Step** | **Text** | **Commands** | **Comment** |
| --- | --- | --- | --- |
| **3.** Thread target sequence bRh onto template B2Ar backbone coordinates. | The sequence of the target GPCR was then placed onto the helical backbone coordinates of each template structure. | rosetta_tools/protein_tools/scripts/thread_pdb_from_alignment.py --template=2rh1A_clean --target=1u19A --chain=A --align_format=clustal 1u19A.aln 2rh1A_clean.pdb 1u19A_on_2rh1A.pdb | **Input:**  1u19A.aln 2rh1A_clean.pdb  **Output:**   1u19A_on_2rh1A.pdb |

**4. Rebuild missing density**

| **Step** | **Text** | **Commands** | **Comment** |
| --- | --- | --- | --- |
| **4A.** Generate secondary structure prediction, constraint file and fragments for bRh. |  | **Secondary structure- Jufo9D:** <http://meilerlab.org/index.php/servers/show?s_id=5> **Secondary structure- PSIPRED:** <http://bioinf.cs.ucl.ac.uk/psipred/> **Transmembrane span prediction based on Jufo9D:** perl scripts/jufo9d_span.pl 1u19A.jufo9d > 1u19A.span  **Disulfide bond constraint file:** Create file that lists residue number of cysteine residues predicted to disulfide bond according to the alignment with the template. **Fragment files:**  http://www.robetta.org Check for exclusion of bRh from the fragment database. Not included due to file size restrictions. | **Input:**  1u19A.fasta  **Output:**   1u19A.jufo_ss, 1u19A.psipred_ss2, 1u19A.span, 1u19A.disulfide, aa1u19A03_05.200_v1_3, aa1u19A09_05.200_v1_3 |
| **4B**. Rebuild missing density caused by gaps in the sequence alignment. | Any missing density and variable loop regions were constructed using the ab initio cyclic coordinate descent protocol in Rosetta. | **Generate loops file:** In this case, the loop definitions will span regions where gaps were located in the sequence alignment. List the residue numbers in the loop file as shown in 1u19A_on_2rh1A.loops. **Generate options file:** List the desired options for rebuilding loop regions in an options file as shown in ccd_initial.options. **Run loop building:** rosetta_source/bin/loopmodel.linuxgccrelease @ccd_initial.options -database rosetta_database | **Input:**  ccd_initial.options, 1u19A_on_2rh1A.pdb, 1u19A.span, 1u19A.disulfide, 1u19A_on_2rh1A.loops, aa1u19A09_05.200_v1_3, aa1u19A03_05.200_v1_3   **Output:**  200 models of 1u19A from 2rh1A template with missing density rebuilt, for example: 1u19A_on_2rh1A_initial.pdb |

**5. Rebuild ECL 1,2 and 3**

| **Step** | **Text** | **Commands** | **Comment** |
| --- | --- | --- | --- |
| **5A**.  Construct comparative model by rebuilding loop regions in Rosetta with CCD. | Extracellular loops were extensively rebuilt using both the cyclic coordinate descent loop closure method described above and the kinematic loop closure method described below. | **Generate loops file:** The loop definitions span the region between transmembrane helices. List the residue numbers for extracellular loops in the loop file as shown in 1u19A.loops. **Generate options file:** List the desired options for rebuilding loop regions in an options file as shown in ccd.options. **Run loop building:** rosetta_source/bin/loopmodel.linuxgccrelease @ccd.options -database rosetta_database | **Input:**  ccd.options, 1u19A_on_2rh1A_initial.pdb, 1u19A.span, 1u19A.disulfide, 1u19A.loops, aa1u19A09_05.200_v1_3, aa1u19A03_05.200_v1_3  **Output:**  1000 models of 1u19A from 2rh1A template with ECLs rebuilt, for example:  1u19A_rmsd01.pdb |
| **5B**.  Construct comparative model by rebuilding loop regions in Rosetta with KIC. | Extracellular loops were extensively rebuilt using both the cyclic coordinate descent loop closure method described above and the kinematic loop closure method described below. | **Generate loops file:** The loop definitions span the region between transmembrane helices. List the residue numbers for extracellular loops in the loop file as shown in 1u19A.loops. **Generate options file:** List the desired options for rebuilding loop regions in an options file as shown in kic.options. **Run loop building:** rosetta_source/bin/loopmodel.linuxgccrelease @kic.options -database rosetta_database | **Input:**  kic.options, 1u19A_on_2rh1A_initial.pdb, 1u19A.span, 1u19A.disulfide, 1u19A.loops  **Output:**  1000 models of 1u19A with ECLs rebuilt, for example: 1u19A_rmsd01.pdb |

**6. Evaluate comparative models by clustering by full-receptor RMSD and knowledge-based pocket residue filter**

| **Step** | **Text** | **Commands** | **Comment** |
| --- | --- | --- | --- |
| **6A.** Analyze results by clustering top ten percent of comparative models by full receptor RMSD. | As Rosetta energy units demonstrated a correlation to the quality of comparative model when compared to crystal structures using root mean square deviation (RMSD) on C-alpha atoms, the top ten percent of models by energy were chosen for further evaluation via clustering. To further rank the models, pairwise RMSDs were calculated between the models and bcl::Cluster (Alexander et al, 2011) was used to identify clusters of similar loop conformations with a cluster radius of 3 angstroms. | **Filter for the top ten percent of models by energy:**  Rosetta energy units incorporating the implicit membrane potential for each model are found in the *.out file.  **Generate table of pairwise RMSD values:**  bcl::PDBCompare was used to generate a table of pairwise RMSD values between comparative models. Download the bcl software suite at (the license is free for non-commercial users).  <http://www.meilerlab.org/index.php/bclcommons/show/b_apps_id/12>  bcl.exe PDBCompare -quality RMSD -atoms CA -pdb_list 1u19A_models.ls -aaclass AACaCb -prefix 1u19A_10percent_  **Cluster models by RMSD:**  bcl.exe Cluster -distance_input_file 1u19A_10percent_RMSD.txt -input_format TableLowerTriangle -output_format Rows Centers -output_file cluster3_1u19A -linkage Average -remove_internally_similar_nodes 3 | **Input:**  PDB files for top ten percent of 1u19A comparative models by Rosetta energy and names of those PDB files in a list called 1u19A_models.ls  **Output:**  1u19A_10percent_RMSD.txt, cluster3_1u19A.Centers, cluster3_1u19A.Rows |
| **6B.** Analyze results by filtering comparative models with a knowledge-based filter. | To interrogate and avoid sampling of non-native flexibility of pocket residues we constructed a knowledge-based filter. Pocket residues were defined as a residue in any GPCR that had at least a 4 Å distance to the ligand in the crystal structure. | **Calculate the minimum distance to any alignment equivalent position in any GPCR:**  For all pocket residues the minimum distance to any sequence alignment equivalent residue in any GPCR is determined with PyMOL.  scripts/evaluate_score_vs_pocket_rmsd/ 01_make_distances.csh  scripts/evaluate_score_vs_pocket_rmsd/ 02_filter_models.py | **Input:**  Structures to be filtered should be in: scripts/evaluate_score_vs_pocket_rmsd/ structures/ID/ID_struc_id.pdb  Crystal structures for distance calculations are placed in: crystal_pockets/  The residue numbering of the models must be identical to that of the crystal structures. **Output:**  A list of filtered structures is generated in scripts/evaluate_score_vs_pocket_rmsd/ pdb_lists_filtered/ |

**7. Generate ligand conformations in MOE**

| **Step** | **Text** | **Commands** | **Comment** |
| --- | --- | --- | --- |
| **7.** Create ligand conformations in MOE. | Ligand conformations were generated by MOE (Molecular Operating Environment, Chemical Computing Group, Ontario, Canada) with the MMFF94x force field and Generalized Born solvation model. Energy cutoffs for ligand conformations were dependent on the number of rotatable bonds: 3 kcal/mol for 1-6 rotatable bonds, 5 kcal/mol for 7-9 rotatable bonds and 7 kcal/mol for 10-12 rotatable bonds (Perola and Charifson, 2004). | **Generate ligand conformations in MOE:**  See MOE operating guide. LowModeMD with the MMFFx94 force field and Generalized Born solvation model was used to generate conformations within the specified energy cutoff. The ligand conformations were then saved as an .sdf file for conversion to .pdb and .params files for Rosetta. **Convert .sdf file of ligand conformations to .pdb and .params file for Rosetta input:**  rosetta_source/src/python/apps/public/molfile_to_params.py -n 1u19A -p 1u19A 1u19A.sdf  Combine all individual ligand conformations in pdb format to a file called 1u19A_confs.pdb.  Add the line “PDB_ROTAMERS 1u19A_confs.pdb” to the bottom of the 1u19A.params file. | **Input:**   ligand coordinates in mol format: 1u19A.sdf  **Output:**  1u19A.params, 1u19A_confs.pdb |

**8. Dock ligand into comparative models**

| **Step** | **Text** | **Commands** | **Comment** |
| --- | --- | --- | --- |
| **8A.** Generate input files necessary for docking with Rosetta Scripts. | Each ligand was allowed to sample docking poses in a 5 Å radius from the crystallized binding pose. After a rigid body orientation of the ligand centroid is performed through translation and 1000 cycles of 360 degree rotation, varying conformations of the ligand were tested within the site. During high resolution refinement, six cycles of side-chain rotamer sampling around the ligand were coupled with 0.1 angstrom, 0.05 radian ligand movements simultaneously in a Monte Carlo simulated annealing algorithm. A final minimization combines side-chain rotamer sampling with backbone torsion angle minimization with harmonic constraints on the C-alpha atoms. | **Prepare input pdb files:** Align the comparative model for docking to the crystal structure 1u19A_clean.pdb. Copy one ligand conformation from the 1u19A_confs.pdb file to the bottom of the pdb file of the starting model, 1u19A_cluster01_01.pdb. Save as 1u19A_cluster01_01_ligand.pdb.  **Prepare options file for docking:** List the desired options for docking in an options file as shown in dock.options.  **Prepare XML file for docking:** List the desired specifications for docking in an options file as shown in dock.xml. | **Input:**  1u19A_clean.pdb, 1u19A_confs.pdb, 1u19A_cluster01_01.pdb    **Output:**  1u19A_cluster01_01_ligand.pdb, dock.options, dock.xml |
| **8B.** Dock ligand within bRh comparative models. | For each ligand, over 2,000 docked complexes were generated. | rosetta_source/bin/rosettascripts.linuxgccrelease @dock.options -database rosetta_database | **Input:**   1u19A_cluster01_01_ligand.pdb, 1u19A.params, dock.xml, dock.options   **Output:**  1000 models of retinal bound to bRh, for example:  1u19A_cluster01_01_ligand_011u19A_cluster01_01_ligand_0001.pdb |

**9. Analyze results by clustering binding modes by ligand RMSD**

| **Step** | **Text** | **Commands** | **Comment** |
| --- | --- | --- | --- |
| **9.** Analyze results by clustering binding modes by ligand RMSD. | All models were evaluated using clustering on pairwise RMSD calculated for the ligand coordinates using bcl::Cluster with a 3 angstrom cutoff. The lowest energy binding modes of the largest clusters were chosen as the best representative of the docking study. | **Align PDBs :**  Use PyMOL to align receptor backbone coordinates.  **Extract lines for ligand coordinates into an .sdf file:**  /scripts/rmsd.tcsh *.pdb  **Generate table of pairwise RMSD values:**  bcl::ScoreSmallMolecule was used to generate a table of pairwise RMSD values between ligand coordinates. Download the bcl software suite at (the license is free for non-commercial users).  <http://www.meilerlab.org/index.php/bclcommons/show/b_apps_id/12>  bcl.exe ScoreSmallMolecule all.sdf output.sdf -comparison RMSD  **Cluster models by RMSD:**  bcl.exe Cluster -distance_input_file 1u19A_ligand.cluster.mat -input_format TableLowerTriangle -output_format Rows Centers -output_file cluster3_1u19A_ligand -linkage Average -remove_internally_similar_nodes 3 | **Input:**  PDB files for 1u19A docked models.  **Output:**  all.sdf, 1u19A_ligand.cluster.mat, cluster3_1u19A_ligand.Centers, cluster3_1u19A_ligand.Rows |
